# Mesenchymal stromal cells facilitate resolution of pulmonary fibrosis by miR-29c and miR-129 intercellular transfer

**DOI:** 10.1038/s12276-023-01017-w

**Published:** 2023-07-03

**Authors:** Basalova Nataliya, Arbatskiy Mikhail, Popov Vladimir, Grigorieva Olga, Vigovskiy Maksim, Zaytsev Ivan, Novoseletskaya Ekaterina, Sagaradze Georgy, Danilova Natalia, Malkov Pavel, Cherniaev Andrey, Samsonova Maria, Karagyaur Maxim, Tolstoluzhinskaya Anastasiya, Dyachkova Uliana, Akopyan Zhanna, Tkachuk Vsevolod, Kalinina Natalia, Efimenko Anastasiya

**Affiliations:** 1 Institute for Regenerative Medicine, Medical Research and Education Center, Lomonosov Moscow State University, Moscow, Russian Federation; 2 Faculty of Medicine, Lomonosov Moscow State University, Moscow, Russian Federation; 3 Department of Clinical Pathology, Medical Research and Education Centre, Lomonosov Moscow State University, Moscow, Russian Federation; 4 Division of Fundamental Medicine of Federal State Budgetary Institution “Pulmonology Scientific Research Institute under Federal Medical and Biological Agency of Russian Federation”, Moscow, Russian Federation; 5 Research Institute of Human Morphology, Moscow, Russian Federation

**Keywords:** Mechanisms of disease, Differentiation, miRNAs, Translational research

## Abstract

To date, pulmonary fibrosis remains an unmet medical need. In this study, we evaluated the potency of mesenchymal stromal cell (MSC) secretome components to prevent pulmonary fibrosis development and facilitate fibrosis resolution. Surprisingly, the intratracheal application of extracellular vesicles (MSC-EVs) or the vesicle-depleted secretome fraction (MSC-SF) was not able to prevent lung fibrosis when applied immediately after the injury caused by bleomycin instillation in mice. However, MSC-EV administration induced the resolution of established pulmonary fibrosis, whereas the vesicle-depleted fraction did not. The application of MSC-EVs caused a decrease in the numbers of myofibroblasts and FAPa^+^ progenitors without affecting their apoptosis. Such a decrease likely occurred due to their dedifferentiation caused by microRNA (miR) transfer by MSC-EVs. Using a murine model of bleomycin-induced pulmonary fibrosis, we confirmed the contribution of specific miRs (miR-29c and miR-129) to the antifibrotic effect of MSC-EVs. Our study provides novel insights into possible antifibrotic therapy based on the use of the vesicle-enriched fraction of the MSC secretome.

## Fibrosis: stem cell–secreted vesicles contain anti-scarring RNA molecules

Tiny regulatory RNAs found within membrane-bound vesicles released by fat-derived stem/stromal cells help to resolve lung scarring in a mouse model of pulmonary fibrosis. Nataliya Basalova from Lomonosov Moscow State University in Russia and coworkers observed that molecules secreted by human adult stem/stromal cells help to reduce lung fibrosis in mice with established disease but do not prevent scarring early in the disease process. The researchers traced the beneficial effects to a specific pair of microRNAs contained within vesicles that the stem cells release into their environment. These microRNAs cause scar-forming cells to return to a more flexible state, leading to healthier lung function. Drugs that take advantage of these healing properties could help treat fibrosis, a feature of many chronic diseases, in the lungs and other organs.

## Introduction

Severe and chronic pneumonia, including damage after pulmonary infection (e.g., SARS-CoV-2), autoimmune disease, or idiopathic causes, often results in pulmonary fibrosis, which ultimately impairs respiratory function, affects the quality of life, and shortens lifespan. A hallmark of fibrosis is the replacement of airy alveoli by stromal cells, which produce excessive amounts of extracellular matrix (ECM). A “fire unit” of fibrosis development and progression is a fibrotic focus consisting of myofibroblasts of multiple origins, which deposit misfolded collagens I, III, IV, V, and VI, as well as EDA-fibronectin. Myofibroblasts have stress fibers formed by α-smooth muscle actin (αSMA) and demonstrate an increased ability to contract the ECM. Actively proliferating and invasive fibroblasts are positive for fibroblast activation protein alpha (FAPa^+^) and other fibroblast precursors appear on the fibrotic focus periphery, which is responsible for foci expansion and merging^1^, promoting fibrosis progression and subsequent respiratory disturbance.

Several investigators have attempted to harness pathological fibroproliferative reactions using mesenchymal stromal/stem cells (MSCs). To date, experimental models have demonstrated that the antifibrotic effect of MSCs is mainly mediated by their specific secretory activity^2–5^. Thus, MSCs can suppress fibrogenesis due to the action of soluble paracrine factors (MSC-SF) and extracellular vesicles (MSC-EV), which carry various regulatory non-coding RNAs, including microRNA (miR)^6,7^, and contribute to the destruction of ECM through matrix metalloproteinase secretion. These data provide a basis for initiating clinical studies using MSC-EVs as a therapeutic agent for lung injuries, particularly SARS-CoV-2-induced pneumonia (NCT04491240 and NCT04276987). Previously, we demonstrated that the MSC secretome fraction enriched with MSC-EVs was able to suppress fibroblast differentiation into myofibroblasts and induce dedifferentiation^8^. Therefore, this mechanism can govern the antifibrotic action of the MSC secretome.

In this study, we evaluated the ability of the MSC secretome, divided into fractions enriched with MSC-EVs or MSC-SFs, to prevent pulmonary fibrosis following bleomycin-induced damage or facilitate the resolution of established pulmonary fibrosis. Our data indicate that MSC-EV administration attenuates fibrotic progression and induces the resolution of established pulmonary fibrosis, whereas MSC-SF does not. We also demonstrated the possible molecular and cellular mechanisms underlying these effects. Furthermore, we showed a decrease in the numbers of myofibroblasts and FAPa^+^ progenitors within the fibrotic lung tissue without an effect on their apoptosis 2 weeks after intratracheal administration of MSC-EVs. Based on our previous data, we suggest that myofibroblasts likely dedifferentiate owing to specific antifibrotic miR transfer by MSC-EVs. In this study, we confirmed the contribution of specific miRs, miR-29c and miR-129, to the antifibrotic action of MSC-EVs.

## Methods

### Cell culture

hTERT immortalized adipose-derived mesenchymal stem cells (hTERT-MSC, ASC52telo (ATCC® SCRC-4000™)), hTERT-MSCs with Crispr–Cas9 miR-29с knockdown and hTERT-MSCs with Crispr–Cas9 nonsense knockdown (biobank of the Institute for Regenerative Medicine, Lomonosov MSU (https://human.depo.msu.ru)) were cultured in Advance Stem Cell Basal Medium with Advance Stem Cell Growth Supplement (HyClone). All experiments were conducted using cells from passages 15–25.

Human lung fibroblast (HLF)-210 cells were generously provided by the University of Manchester (United Kingdom). Human dermal fibroblasts (HDFs), murine lung fibroblasts (MLFs), murine adipose-derived mesenchymal stem cells (mMSCs), human umbilical endothelial cells (HUVECs), and A549 epithelial cells were obtained from the biobank of the Institute for Regenerative Medicine, Lomonosov MSU (https://human.depo.msu.ru). All cell lines were cultured in Dulbecco’s modified Eagle’s medium with low glucose (DMEM-LG) with 10% fetal bovine serum (FBS), and 1% penicillin–streptomycin (all from Gibco), and the HUVECs were cultured in EGM-2 (Lonza). All experiments were conducted using cells within 10 passages. All procedures performed using tissue samples from patients were in accordance with the Declaration of Helsinki and approved by the ethic committee of Lomonosov Moscow State University (IRB00010587), protocol #4 (2018).

### Conditioned medium harvesting and fractioning

The MSC-EV fraction and EV-depleted fraction (MSC-SF) were isolated from hTERT-MSC- and mMSC-conditioned medium (CM) as described earlier^8^. The MSC-EV fraction was also isolated using Amicon gradient ultracentrifugation (Optima XPN-100 Ultracentrifuge)^9^. All samples were stored at −80 °C. The particle size and concentration of MSC-EVs were analyzed via nanoparticle tracking analysis (NTA; ZetaView, Particle Metrix), and the morphology was visualized via transmission electron microscopy (TEM). Exosomal markers in MSC-EVs or MSC lysates were evaluated by immunoblotting.

For the removal of RNA from the hMSC-EVs, the protocol described by Otsuru et al. was employed^10^. Briefly, the isolated and concentrated hMSC-EVs were treated in 1 mL of phosphate-buffered saline (PBS, Paneco) containing 5 U of RNase A (Thermo Fisher Scientific) at 37 °C for 3 h to remove the RNA inside the EVs or for 30 min to enable RNA depletion in the medium and outer membrane of the EVs. Subsequently, RiboLock RNase Inhibitor (Thermo Fisher Scientific) was used to inhibit RNase A activity within 10 min. The treated hMSC-EVs were isolated from suspension on an Amicon filter (1000 kDa) as described above. Analysis of the length distribution of nucleic acid fragments within native EVs or EVs after RNase treatment was performed using TapeStation (Agilent).

### Fibroblast differentiation in vitro models

For induction of fibroblast-to-myofibroblast differentiation, three fibroblast lines were used (HLF, HDF, and MLF). The cells were seeded in plates (1.5 × 10^4^ cells/cm^2^), grown for 1 day, and serum-deprived overnight in DMEM-LG. Then, fresh DMEM-LG was applied together with 5 ng/mL TGFβ (R&D Systems) and hMSC-EVs or mMSC-EVs (1.75 × 10^4^ part/cells). All plates were placed in a CO_2_ incubator at 37 °C and analyzed after 4 days.

For induction of myofibroblast-to-fibroblast dedifferentiation, HDFs were seeded (7.5 × 10^3^ cells/cm^2^), grown for 1 day, and serum-deprived overnight. Subsequently, fresh DMEM-LG with 5 ng/mL TGFβ was added to the cells for 4 days. Then, the cells were thoroughly washed with Hanks’ buffer solution, and hMSC-EVs (1,75 × 10^4^ part/cells) or DMEM-LG as a control medium was added. The cells were placed in a CO_2_ incubator at 37 °C and analyzed after 4 days.

For analysis, the cells were fixed with 4% paraformaldehyde in PBS for 10 min at room temperature (RT) and permeabilized with 0.1% Triton X-100 for 10 min. After blocking with 10% normal goat serum on 1% bovine serum albumin (BSA) (Abcam), the cells were incubated with an antibody against αSMA (Abcam; ab32575) or rabbit IgG (Santa Cruz Biotechnology; NSC-2025) overnight at 4 °C and further stained with secondary antibodies conjugated with Alexa Fluor-488 (Invitrogen, A11037) for 1 h at RT in the dark. The nuclei were counterstained with DAPI (Sigma‒Aldrich, D9542).

### microRNA level evaluation via real-time quantitative PCR and bioinformatic analysis

Total RNA containing miRs was isolated from mouse lung tissue and purified using (https://norgenbiotek.com/product/total-rna-purification-kit) the Total RNA Purification Kit (Norgen, 17250) according to the manufacturer’s protocols. The RNA was quantified and qualified using the NanoDrop spectrophotometer (Thermo Fisher Scientific) by a 260/230-nm ratio. Samples with A260/280 values from 1.9 to 2.1 were used for further analysis. Reverse transcription was performed using the miScript II RT Kit (Qiagen) according to the manufacturer’s protocol. Real-time PCR was performed using the miScript SYBR Green PCR Kit (Qiagen). Specific commercial primers (Qiagen) for hsa-miR-21, hsa-miR-29c, and hsa-miR-129 were constructed in Primer-BLAST and OligoArchitect primers for housekeeping RNA (RNU6, CGCAAGGATGACACGCAAAT, Evrogen or SNORD96, Qiagen). The expression levels of microRNAs were calculated relative to those of housekeeping RNA using the comparative ΔCT method.

In the miRNet database, 2055 targets were identified for hsa-miR-29c-3p, and 1111 targets were identified for hsa-miR-129-5p. The number of common targets was 227, of which 57 were directly related to myofibroblast dedifferentiation. The list of genes involved in myofibroblast dedifferentiation was made using literature sources (Google Scholar). Commonly predicted microRNA targets were analyzed using the miRNet-service.

Raw data of MSC-EV RNA sequencing are available as PRJNA592301 (NCBI/SRA).

### Western blotting and dot blotting

Cells or EVs were lysed in 2× Laemmli buffer (Bio-Rad Laboratories). The protein in the lysate was quantified using the micro-BCA protein assay (Thermo Fisher Scientific), electrophoresed in SDS‒PAGE gel, and transferred to the PVDF membrane. The membrane was incubated with primary antibodies against αSMA (ab32575, Abcam), α-actinin 1 (sc-17829, Santa Cruz Biotechnology), α-actinin 4 (sc-393495, Santa Cruz Biotechnology), α-actinin 2 (A7732, Sigma), GAPDH (sc-32233, Santa Cruz Biotechnology) or exosome markers (EXOAB-KIT-1, System Biosciences; Alix, ab117600, Abcam; non-EV marker-H2Ax, AF2288, R&D) overnight at 4 °C.

The quantitative analysis of collagen I was carried out by dot-blot analysis (dot‐ELISA). Lysed cell samples were applied in duplicate at 0.5 μL to a nitrocellulose membrane (Amersham) and air‐dried. The subsequent immunodetection used a Western blotting protocol with the incubation of membranes with primary antibodies against collagen I (ab34710, Abcam).

After TBST washes, the blots were incubated with horseradish peroxidase-labeled secondary antibodies (P-RAMIss, P-RAQIss, P‐GARIss, Imtek) for 1 h. The labeled proteins were visualized with a ChemiDocTMTouch imaging system (Bio-Rad Laboratories) using an enhanced chemiluminescence kit (Pierce). To normalize the obtained values of the protein of interest in the case of dot blot, we used the values of the amount of protein obtained by staining the membrane with Amido black. HEK293 lysates were used as a negative control.

### MSC-EV transfection

hMSC-EV transfection was performed using the Exo-Fect^TM^ Exosome Transfection Kit (EXFT20A-1, System Biosciences) with stabilized miR-antagomirs or a pair of miR-antagomirs (miRCURY LNA inhibitors for microRNAs hsa-miR-29c-3p, miR-21-5p, hsa-miR-129-5p, hsa-miR-92a-3p; miRCURY LNA miRNA Inhibitor Control, Qiagen). The transfected EVs were dissolved in fresh DMEM-LG up to the appropriate concentration, and in vitro or in vivo models were used.

### Biotin-labeled pulldown assay

Biotinylated miR-29c and miR-129 (GenTerra) pulldown assays with target mRNAs were performed as described earlier^11^. Briefly, 1 × 10^6^ HDFs were seeded in 10 cm dishes in duplicate a day before transfection. The next day, 3’ biotin-labeled control miR (5′UCACCGGGUGUAAAUCAGCUUG-3′-biotin) or biotin-labeled miRs were transfected in complex with HiPerfect (Qiagen) at a final concentration of 50 nM. After 36 h, whole cell lysates were harvested, mixed with precoated Dynabeads MyOne Streptavidin-C1 (65001, Invitrogen, 50 μl/sample), and incubated for 2 h at 4 °C on a rotator. Beads were then pelleted down to remove unbound materials at 4 °C for 1 min, 5 K rpm, and washed five times with 500 μl of ice-cold lysis buffer (pulldown fraction). Cell lysates were used as input controls.

RNA isolation and PCR analysis were performed according to previously published protocols^8^.

The miR enrichment was calculated as follows:

Biotin-miR pulldown for collagen type I mRNA/biotin-scramble pulldown for collagen type I mRNA = X.

Biotin-miR input for collagen type I/Biotin-scramble input for collagen type I = Y.

Fold binding = X/Y.

Because the expression of the target for the biotin-scramble pulldown sample was not detected, to calculate expression in the microRNA-29 and 129 groups, we used the value of the maximal PCR cycle (50).

### Bleomycin-induced pulmonary fibrosis model in mice

The work was performed on 9- to 20-week-old male C57BL/6 mice (Puschino) weighing 25 ± 1 g. Animal housing and research procedures were conducted in compliance with Directive 2010/63/EU and approved by the local bioethics committee. All invasive procedures, magnetic resonance imaging (MRI), and IVIS studies were conducted under inhalational anesthesia (Aerrane) supplied by the animal anesthesia system (E-Z-7000 Classic System, E-Z-Anesthesia® Systems): 3.5–4% isoflurane mix with atmospheric air for the induction of anesthesia and 2–2.5% isoflurane/air mix for its maintenance.

For pulmonary fibrosis modeling, 3 U/kg bleomycin (Bleocin, Nippon Kayaku Co., #PN0113322/01) in sterile PBS (30 μL) was injected once intratracheally (transoral instillation^12^). Treatment with MSC-CM components was performed within 24 h (prevention mode) or 14 days (treatment mode) after bleomycin instillation. MSC-CM components (hMSC-EV–6.3 × 10^8^ particles/animals or hMSC-SF, concentrated up to the same volume) or DMEM-LG without Phenol Red (as control) were also administered intratracheally (30 μL). Then, after 21 (prevention mode) or 28 (treatment mode) days, the animals were euthanized with a tenfold dose of anesthetic i.p. injection, and bronchoalveolar lavage (BAL) fluid, blood serum, and tissues were collected for analysis.

### MSC-EV biodistribution

hMSC-EV biodistribution in the lung region of mice was measured via fluorescence imaging (FI). For labeling of hMSC-EVs, two approaches were employed. First, hTERT-MSCs were modified by lentiviral transduction of the CD63-GFP construct and used to collect CD63-GFP MSC-EVs according to the aforementioned protocol. Second, the hMSC-EVs were labeled with the PKH26 Red Fluorescent Cell Linker Kit for General Cell Membranes^13^. We observed that the directly labeled EVs exhibited a higher and brighter fluorescence but provided a higher background than the labeled EVs produced by cells^14^. Healthy mice were injected intravenously or intratracheally with 30 µL of labeled hMSC-EVs or with an equal volume of PBS. A total of 16 healthy mice were treated intravenously or intratracheally with labeled hMSC-EVs and then sacrificed after 1 h (*n* = 4), 24 h (*n* = 4), and 3 days (*n* = 4). Four mice received the same amount of labeled hMSC-EVs 2 weeks after bleomycin application.

The mice were anesthetized with 2.5% isoflurane, and images were captured in the posterior position 15 min, 60 min, 24 h, and 3 days post-EV injection using an IVIS Spectrum CT (PerkinElmer). Healthy mice treated with PBS were used as blank controls for the fluorescence signal. The fluorescence signal was quantified in the lung region and the abdominal area in regions of interest (ROI) drawn freehand. The relative mean fluorescence intensity of each ROI was obtained by subtracting the mean fluorescence intensity of the corresponding ROI on the control mouse from the measured mean fluorescence intensity. At the end of the experiments (1 h, 24 h, and 3 days post-EV injection), the mice were sacrificed, and the dissected tissues (lungs, brain, spleen, liver, adipose tissue, and bone marrow) were frozen in Tissue-Tek, sliced and analyzed via fluorescence microscopy.

### Magnetic resonance imaging

MRI was performed using the ClinScan 7 T device (Bruker Biospin, USA). All mice underwent MRI at 0, 7, 14, and 27 days from the start of the experiment. For MRI images, the animals were anesthetized with an oxygen/isoflurane air mixture (98%/2%). The vital activity was monitored using a respiratory cycle monitor. Lung imaging was performed in the T2-weighted mode with suppression of the signal from the adipose tissue using the Turbo Spin Echo sequence with the following parameters: TR, 1175 ms; TE, 55 ms; echo train length, 8; FOV, 42 × 60 mm; and base resolution, 216 × 384. The imaging analysis was conducted using ImageJ software. We only used images of slices that passed through the chest in the frontal plane (*n* = 3–10 for each animal).

### BAL analysis

For collection of BAL fluid, the contents of the bronchi and lungs were washed 5 times with 0.5 ml of PBS with 10% FBS. After BAL suspension centrifugation, the cell pellets were suspended in 100 μL of PBS, and a 10 μL suspension was placed on a glass slide in duplicate and then dried for 24 h. The preparations were stained with a Differential Quik Stain Kit (Electron Microscopy Sciences) according to the protocol, embedded in the synthetic polymer (CS703, Dako), and analyzed using a Leica DM600Β microscope. Ten random fields were examined at a magnification of 20× for each preparation. The numbers of lymphocytes, neutrophils, and macrophages were counted^15^. The cell composition was expressed as a percentage of the total number of cells in the sample.

The remaining cells were analyzed using a flow cytometer for markers of major hematopoietic cells (BioLegend, 133305). A mixture of isotypic antibodies was used as a control. The percentage of positive cells in the total cell population was measured.

The cell-free part of the BAL was analyzed via ELISAs for the content of the proinflammatory factors MCP-1 (BD Biosciences) and IL-8 (MyBioSource) using commercial kits.

### Histological and immunostaining studies

Samples of lung tissue fixed with 10% neutral formalin were embedded in paraffin. The paraffin Section (1 μm) was dewaxed, stained, embedded in the synthetic polymer (CS703, Dako) or Aqua-PolyMount for IHC, and analyzed blindly. For analysis, sections were stained with hematoxylin and eosin (Dako), Picrosirius Red (collagen formation, 2–3 serial sections for each animal, 6–10 fields of view on the section were evaluated), Masson staining (severity of fibrotic changes according to the Ashcroft scale^16^, Artisan, Dako), and a TUNEL kit (cells with activated death program, Abcam, ab206386).

For the IHC analysis, antigens on sections were unmasked in citrate or TE buffer at 95 °C for 20 min. For reduction of the background fluorescence, the samples were treated with 50 mM ammonium acetate, and then, the nonspecific site was blocked with the normal 10% serum of the animal donor of the secondary antibodies (Abcam) in 1% BSA. For detection of the targets, primary antibodies against CD163 (Abcam; ab182422), FAPa (Bioss, Inc.; bs-5758R), αSMA (Abcam; ab5694, or BioLegend; 904601), and CD90 (Invitrogen; PA5-80127) overnight. Detection of the primary antibodies was performed using secondary antibodies conjugated with Alexa Fluor-488 (A11034, A11001; Invitrogen), Alexa Fluor-594 (A21203, A11032; Invitrogen), and Alexa Fluor Plus-647 (A32733, A32728; Invitrogen). The nuclei were counterstained with DAPI. Microscopic examination was conducted on a Leica DMi8 microscope with a Leica DFC 7000 T camera (Leica Microsystems) using representative fields of view to obtain images. For further analysis and plotting, 1-2 sections were used for each animal, and 5–9 microphotographs were obtained for each section. Image processing and analysis were performed using LasX and FiJi software. Image panels were assembled in the programs FiJi and GNU Image Manipulation Program (GIMP).

### Statistical analysis

Statistical analysis was conducted using GraphPad Prism software. Experimental data are expressed as the median (±25, 75 percentiles, ±min and max). The Kruskal–Wallis *H*-test with the Dunn test was employed for multiple comparisons. Differences were considered significant when *P < 0.05.

## Results

### The components of the MSC secretome do not prevent pulmonary fibrosis development when applied during the acute injury period

**Fig. 1:**
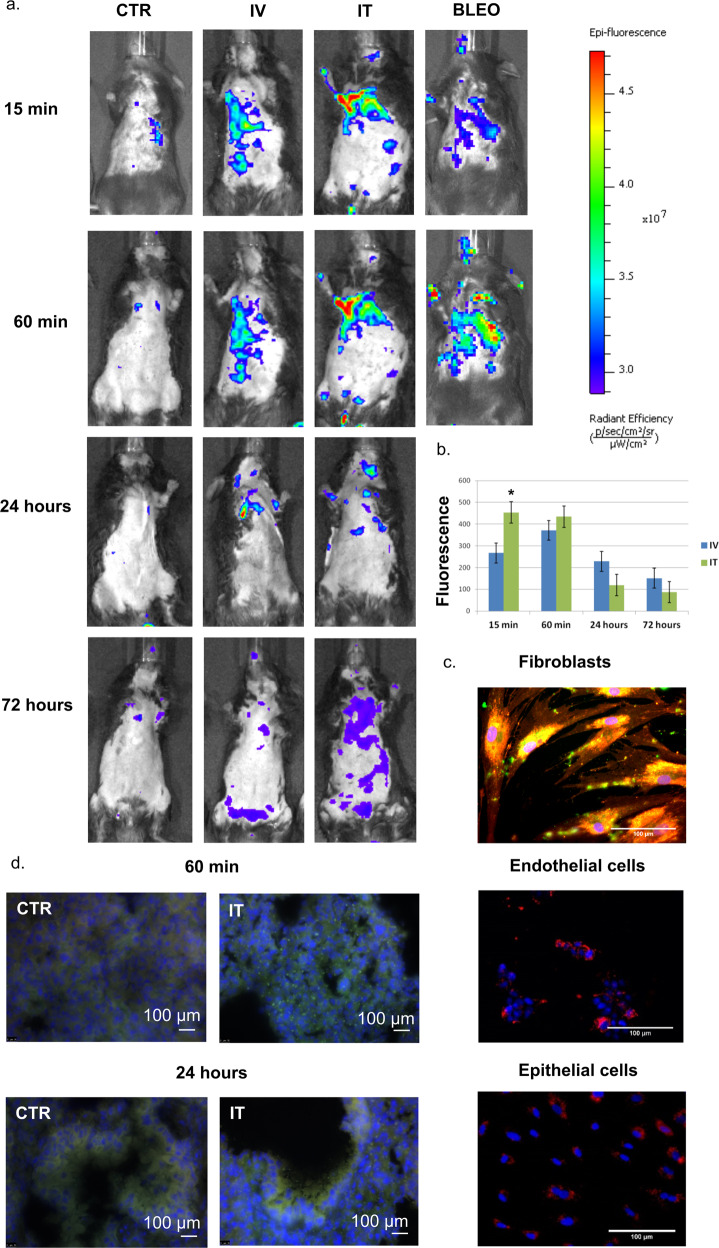
MSC-EV biodistribution in the lung region measured via fluorescence imaging (FI). **a** Representative FI images acquired in the posterior position in healthy mice treated intravenously (IV) or intratracheally (IT) with labeled hMSC-EVs or with an equal volume of phosphate-buffered saline (PBS) (CTR). Additional FI images show mice following the IT administration of bleomycin 2 weeks before treatment with the same amount of labeled hMSC-EVs (BLEO). A total of 16 healthy mice were treated IV or IT with labeled hMSC-EVs; four mice received the same amount of labeled hMSC-EVs 2 weeks after bleomycin application. **b** Quantification of fluorescence intensity in regions-of-interest (ROI) drawn free hand in the lung region, expressed as the average radiance efficiency normalized to the CTR group. *P < 0.05. **c** Uptake of labeled hMSC-EVs (green/red) by fibroblasts and endothelial (HUVEC) and epithelial (A549) cells after 48 h of incubation in vitro. **d** Visualization of labeled hMSC-EVs (green) in the epithelial cells and surrounding stromal cells on lung cryosections 1 and 24 h after intratracheal administration; cell nuclei are labeled with DAPI.

We first tested the ability of MSC secretome components to prevent pulmonary fibrosis development using a murine model of bleomycin-induced lung injury^17^. The MSC-EV fraction was characterized according to the established criteria recommended by the International Society for Extracellular Vesicles (MISEV2018^18^) (Supplementary Fig. 1). In vivo analysis of fluorescently labeled hMSC-EV biodistribution using IVIS revealed some advantages of the intratracheal administration route; thus, fluorescent labels were detected in the lungs immediately after the intratracheal administration of hMSC-EVs and persisted for at least 24 h. Fluorescent particles corresponding to the aggregates of labeled hMSC-EVs were also found in the epithelial cells and surrounding stromal cells on lung cryosections 1 and 24 h after intratracheal administration (Fig. 1).

Using the model of TGFβ-induced fibroblast-to-myofibroblast differentiation, we compared the species-specific antifibrotic effects of MSC-EVs (Supplementary Fig. 2). Thus, we confirmed that the addition of TGFβ to the culture medium increases the number of myofibroblasts with aSMA-positive stress fibers in all three studied lines (HLF-210, MLF, and HDF as a well-characterized control line) in comparison with the non-TGFβ controls. The simultaneous addition of hMSC-EVs with TGFβ inhibited the appearance of aSMA-positive cells in each of the three lines. Notably, the addition of mMSC-EVs had an inhibitory effect only on the MLF model, which may indicate an incomplete homology of MSC-EV composition between different species. Thus, we demonstrated that the obtained hMSC-EV samples met the international criteria for EVs, can be absorbed and accumulate in murine lung tissue, and can also affect the differentiation of MLFs in vitro, comparable to mMSC-EVs, allowing them to be used for further experiments on animals.

**Fig. 2:**
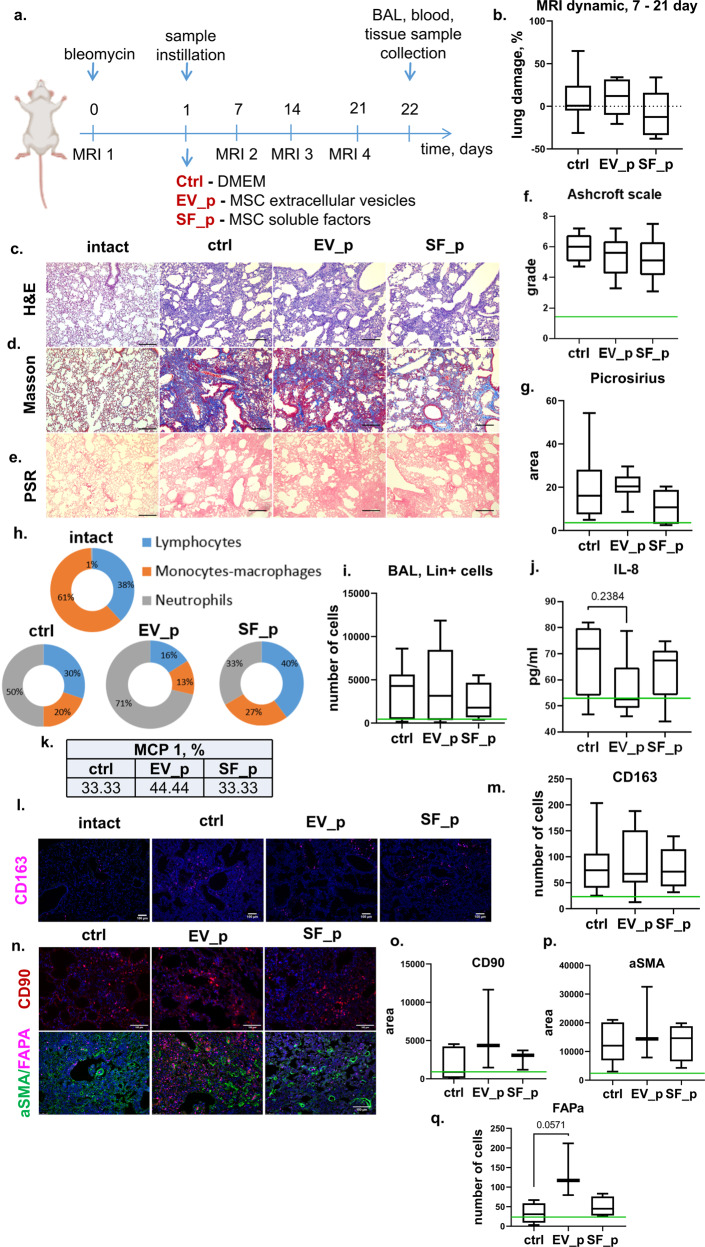
MSC secretome components do not prevent pulmonary fibrosis development when applied during the acute injury period. **a** Schematic design of the prevention of bleomycin-induced pulmonary fibrosis in C57BL/6 mice; Ctrl (DMEM 1 day after bleomycin administration), *n* = 11; EV_p (MSC extracellular vesicles 1 day after bleomycin administration), *n* = 9; SF_p (MSC soluble factors 1 day after bleomycin administration), *n* = 6, *n* = biologically independent animals per group. **b** Quantification of dynamic changes in the lung tissue density measured via MRI. **c**–**e** Representative image of **c** hematoxylin–eosin (H&E), **d** Masson trichrome, and **e** Picrosirius Red (PSR) staining. Scale bar = 100 μm. **f** Quantification of pulmonary fibrosis severity using the Ashcroft scale. The assessment was conducted by two independent blinded experts. **g** Quantification of ECM deposition on PSR staining images. **h** Quantification of immune cell types in BAL smears. The assessment was performed by two independent blinded experts. **i** Quantification of the major hematopoietic cell lineages in the BAL fluid via flow cytometry. **j** Quantification of IL-6 in the BAL using ELISAs. **k** Percentage of animals with a detected rate of MCP-1 in the BAL via ELISAs. **l** Representative images of pulmonary tissue immunocytochemical analysis for CD163. Scale bar = 100 μm. **m** Quantification of CD163-positive cells in pulmonary tissue. **n** Representative images of pulmonary tissue immunocytochemical analysis for CD90 (top panel), aSMA (green), and fibroblast activation protein A (FAPa, red) (bottom panel) expression. Scale bar = 100 μm. **o**–**q** Quantification of CD90- (**o**), aSMA- (**p**), and FAPa- (**q**) positive cells measured by average cell area (**c**, **d**) or number of cells (**e**) in the pulmonary tissue sections, *n* = 4, *n* = biological independent animals per group. The number of analyzed fields of view per sample = 5–9. The green line indicates the median for the intact group.

For analysis of the ability of MSC secretome components to prevent pulmonary fibrosis development, both fractions of the MSC secretome, hMSC-EV, and hMSC-SF, were intratracheally administered the next day after bleomycin application in mice (Fig. 2a). MRI analysis revealed that the bleomycin-treated mice developed pulmonary fibrosis within 3 weeks (Fig. 2b, Supplementary Fig. 3a, b). According to the Ashcroft scale, the degree of pulmonary fibrosis reached 5–7 points (Fig. 2c, d, f) due to increased collagen deposition (Fig. 2e, g). Furthermore, the fibrotic changes in the stroma of the lungs were accompanied by an increase in the lin^+^ leukocyte count in the BAL fluid and a significant shift in the immune cell ratio. Thus, the number of total lin^+^ cells increased 6-fold, and the neutrophil ratio increased by up to 50-fold (Fig. 2h, i). The increased neutrophil numbers correlated well with the elevated level of prominent neutrophil chemoattractant IL-8 (CCL8) in the BAL fluid (Fig. 2j). Furthermore, the number of CD163^+^ macrophages infiltrating the lung stroma increased up to threefold compared with that in intact animals (Fig. 2l, m).

Contrary to our expectation, the administration of MSC secretome components did not prevent bleomycin-induced fibrotic changes, as revealed by MRI and histology (Fig. 2). The amounts of lin^+^ leukocytes in the BAL or CD163^+^ macrophages within the lung stroma also did not change compared with those of the control bleomycin-treated animals. However, the ratio of neutrophils in the BAL fluid was the highest in the mice treated with MSC-EVs (Fig. 2h). This was observed despite the tendency toward a decrease in the IL-8 ratio in the BAL fluid of the mice treated with hMSC-EVs (Fig. 2j). These data indicate that local application of the MSC secretome during the acute injury period neither prevents fibrosis nor diminishes its signs.

### MSC-EVs but not MSC-secreted soluble factors attenuate pulmonary fibrosis and may cause its resolution

**Fig. 3:**
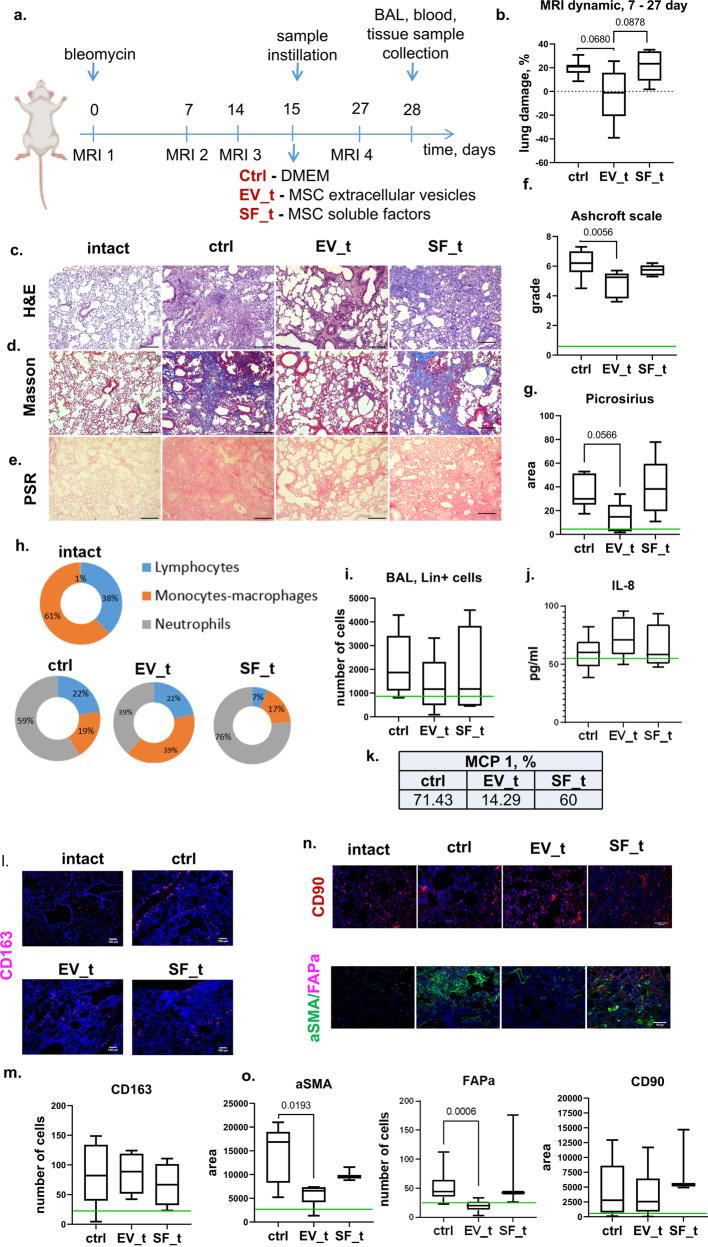
MSC-EVs attenuate pulmonary fibrosis and may cause its resolution. **a** Schematic design of the treatment of bleomycin-induced pulmonary fibrosis in C57BL/6 mice; Ctrl (DMEM 14 days after bleomycin administration), *n* = 11; EV_t (MSC extracellular vesicles 14 days after bleomycin administration), *n* = 9; SF_t (MSC soluble factors 14 days after bleomycin administration), *n* = 6; *n* = biologically independent animals per group. **b** Quantification of dynamic changes in the lung tissue density measured via MRI. **c**–**e** Representative image of **c** hematoxylin–eosin (H&E), **d** Masson trichrome, and **e** Picrosirius Red (PSR) staining. Scale bar = 100 μm. **f** Quantification of pulmonary fibrosis severity using the Ashcroft scale. The assessment was performed by two independent blinded experts. **g** Quantification of ECM deposition on the PSR staining image. **h** Quantification of immune cell types in BAL fluid smears. The assessment was performed by two independent blinded experts. **i** Quantification of the major hematopoietic cell lineages in the BAL fluid via flow cytometry. **j** Quantification of IL-6 in the BAL fluid using ELISAs. **k** Percentage of animals with a detected rate of MCP-1 in the BAL fluid using ELISAs. **l** Representative images of pulmonary tissue immunocytochemical analysis for CD163. Scale bar = 100 μm. **m** Quantification of CD163-positive cells in pulmonary tissue. **n** Representative images of pulmonary tissue immunocytochemical analysis for aSMA, FAPa, and CD90. Scale bar = 100 μm. **o** Quantification of FAPa-positive cells or CD90^+^ and aSMA^+^ areas in pulmonary tissue. The green line indicates the median for the intact group.

Earlier, we demonstrated that MSC secretome components, especially MSC-EVs, can diminish the number of key fibrosis drivers, myofibroblasts, by inhibiting their differentiation and inducing dedifferentiation^8^. Therefore, we analyzed the effect of MSC secretome components on bleomycin-induced pulmonary fibrosis at the remodeling stage, which requires the involvement of myofibroblasts. MSC secretome components were administered 14 days after bleomycin treatment (Fig. 3a). In the control group, 33% of the animals died, and only 11% died after the introduction of hMSC-EVs. In the bleomycin-treated animals, the area of fibrotic damage increased with an average dynamic of +20% during the period from 7 to 28 days following bleomycin administration (Fig. 3b, Supplementary Fig. 3c-d). Lung airiness (alveolar density) in such animals was severely impaired due to the multiple merging fibrotic foci with a large amount of collagen (Fig. 3d). Bleomycin administration led to a significant increase in the number of stromal CD90^+^ cells, mature aSMA^+^ myofibroblasts and activated FAPa^+^ myofibroblast precursors (Fig. 3n-o, Supplementary Fig. 9).

In the mice receiving hMSC-EVs, the area of fibrotic damage decreased with an average dynamic of −40% (Fig. 3b, Supplementary Fig. 3c, d). The alveolar density 14 days after hMSC-EV administration resembled that of intact lungs and was disturbed only by rare fibrotic foci, which were composed of myofibroblasts (Fig. 3c, d). The collagen deposition and overall fibrosis severity were also significantly lower than those in the bleomycin-treated animals (Fig. 3e–g). This change was accompanied by a significant decrease in the numbers of αSMA^+^ myofibroblast and FAPa^+^ myofibroblast precursors (Fig. 3n, o). Moreover, hMSC-EV administration did not reduce the number of stromal CD90+ cells. Through a TUNEL assay, we demonstrated that hMSC-EVs did not affect the number of apoptotic cells (Fig. 5h). We also noted that MCP-1 production and relative neutrophil numbers were lower in the BAL fluid of these animals (Fig. 3h, k). Furthermore, hMSC-EV administration did not significantly affect the leukocyte ratio in the BAL or the density of CD163^+^ macrophages in the lung stroma (Fig. 3i, j, l, m).

In contrast to the beneficial effect of hMSC-EVs, the introduction of hMSC-SFs did not improve the lung structure; 50–70% of the visual field was occupied by merging fibrotic foci (Fig. 3c, d), the lung airiness was severely disturbed, and the overall fibrotic damage score in some samples reached more than 7 points out of 8 possible points according to the Ashcroft scale (Fig. 3f).

These data suggest that the application of hMSC-EVs prevents pulmonary fibrosis progression and induces fibrosis resolution when administered during the remodeling stage. The main targets of hMSC-EVs appear to be fibrotic foci composed of myofibroblasts and myofibroblast progenitors. Because apoptosis and the total number of stromal CD90+ cells were mainly unaffected after hMSC-EV application, the numbers of myofibroblasts and their progenitors could decrease due to changes in their phenotype and secretory activities.

### MSC-EVs cause myofibroblast dedifferentiation by microRNA transfer

EVs can induce changes in target cell phenotype/function via the transfer of multiple RNA species, including regulatory non-coding RNAs. Previously, we demonstrated that hMSC-EVs could inhibit the differentiation of myofibroblasts through the transfer of miRs and analyzed their composition^8^. Based on these data, we selected miRs involved in fibrosis development, namely, miR-21, miR-29c, miR-129, and miR-92a, and tested their ability to affect the phenotype of myofibroblasts.

**Fig. 4:**
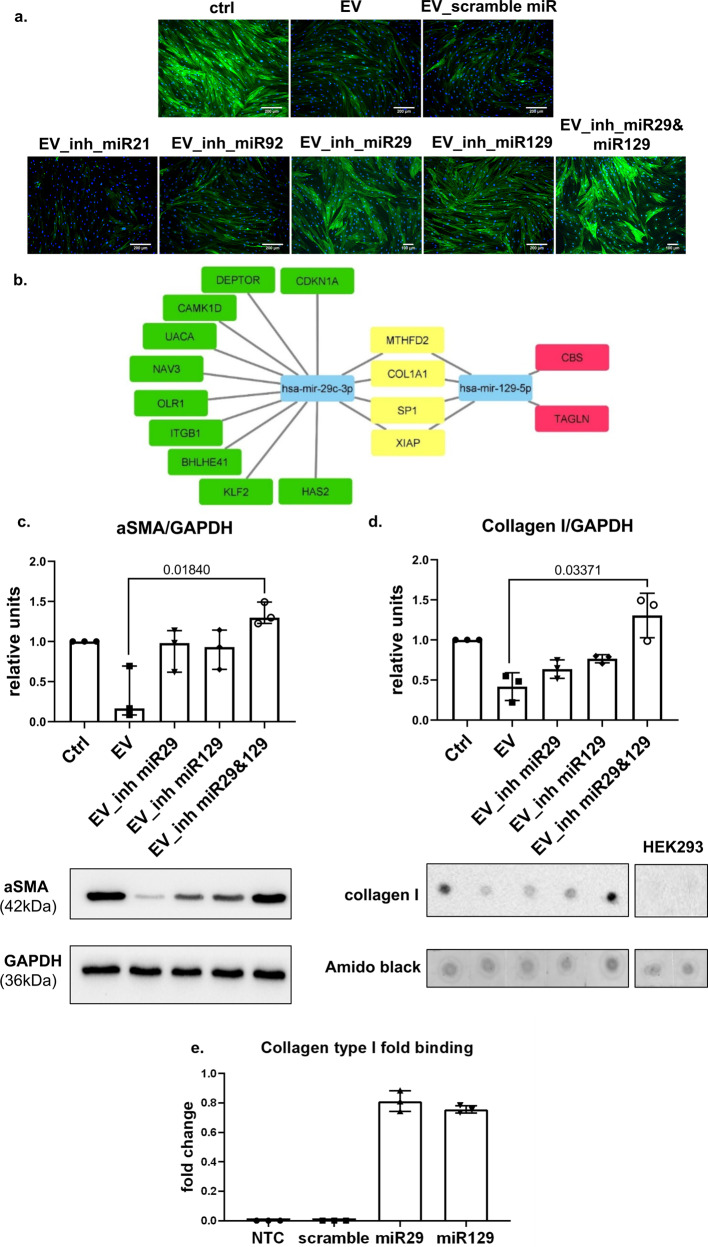
MSC-EVs stimulate myofibroblast dedifferentiation through the transfer of miR-29c and miR-129 in vitro. **a** Representative image of myofibroblast immunocytochemical analysis for αSMA expression. Scale bar = 200 μm. *n* = 4 biological independent experiments. **b** Bioinformatics analysis of specific and common targets for miR-29c and miR-129. **c** Western blot analysis of aSMA. *n* = 3 biological independent experiments. **d** Dot blot analysis of collagen type I. *n* = 3 biologically independent experiments. HEK293 cells were used as a negative control for antibody labeling. **e** Biotinylated microRNA pulldown assay for miR-29c and miR-129 and their target collagen type I. NTC no template control, *n* = 3 biological independent experiments.

We induced fibroblast-to-myofibroblast differentiation via TGFβ treatment and then added hMSC-EVs to differentiated myofibroblasts. Consistent with our previously published data, hMSC-EVs inhibited the expression of αSMA and its incorporation into stress fibers in myofibroblasts, which can be considered dedifferentiation (Fig. 4a, c, d, Supplementary Fig. 4). To evaluate the contribution of RNA transfer by hMSC-EVs to this effect, we removed RNA from hMSC-EVs through RNase treatment, which completely abolished myofibroblast dedifferentiation (Supplementary Fig. 5a top panel, b, c). We showed that RNase treatment for 3 h dramatically reduces the amount of RNA in hEV-MSCs (Supplementary Fig. 5b). However, the number of hEV-MSCs did not significantly decrease, according to NTA analysis (Supplementary Fig. 5c).

To evaluate the contribution of RNA transfer within hMSC-EVs, we treated transfected EVs with RNase for 30 min to remove the RNA from the medium or from the surface of EVs^10^. In addition, we demonstrated that native or transfected EVs retained their biological activity after a short treatment with RNase (Supplementary Fig. 5a, bottom panel).

To evaluate the impact of each selected miR, we transfected hMSC-EVs with specific antagomirs. The inhibition of miR-29c or miR-129 abolished the effect of hMSC-EVs on myofibroblast dedifferentiation, whereas antagomir against miR-21 slightly enhanced this effect. The inhibition of miR-92a did not interfere with the myofibroblast dedifferentiation induced by hMSC-EVs (Fig. 4a).

Bioinformatic analysis revealed several common targets for miR-129 and miR-29c involved in fibrosis development, including XIAP, COL1A1, MTHFD2, and ITGs (Fig. 4b). Furthermore, simultaneous transfection of hMSC-EVs with antagomir to miR-29c and miR-129 led to a complete abolishment of myofibroblast dedifferentiation (Fig. 4a, c). We demonstrated that inhibition of two miRs significantly increased the amount of their common profibrotic target, collagen type I (Fig. 4d). To confirm that miR-29c and miR-129 affect collagen I mRNA and protein expression by direct interaction with its mRNA, we used biotin-labeled miR-29c and miR-129 (Supplementary Tables 1 and 2). The level of collagen I mRNA was markedly elevated in the pulldown material isolated from cells following transfection with biotin-labeled miR-29c and miR-129 compared to the control (Fig. 4e). There was no difference in the levels of 36B4 mRNA.

We assumed that these miRs should have targets that would be directly related to myofibroblast dedifferentiation. Indeed, we found that miR-29c and −129 had 4 targets (vinculin, a-actinins 1, 2, and 4) related to the focal contact formation. We showed that inhibition of miR-29c within hMSC-EVs tended to increase the expression of its targets vinculin and a-actinin 2, whereas the inhibition of miR-129 within hMSC-EVs led to an increase in a-actinin 4 but not a-actinin 1 protein levels in myofibroblasts (Supplementary Fig. 6b, c). We also confirmed our results using hTERT-MSC line with suppressed expression of miR-29c by Crispr-Cas9 (Supplementary Fig. 6a).

Thus, our data indicated that the effect of hMSC-EVs on lung myofibroblasts and fibrotic resolution could be mediated by the transfer of miR-29c and miR-129.

### Transfer of microRNA-29c and microRNA-129 mediates the effects of MSC-EVs on the pulmonary fibrotic resolution

**Fig. 5:**
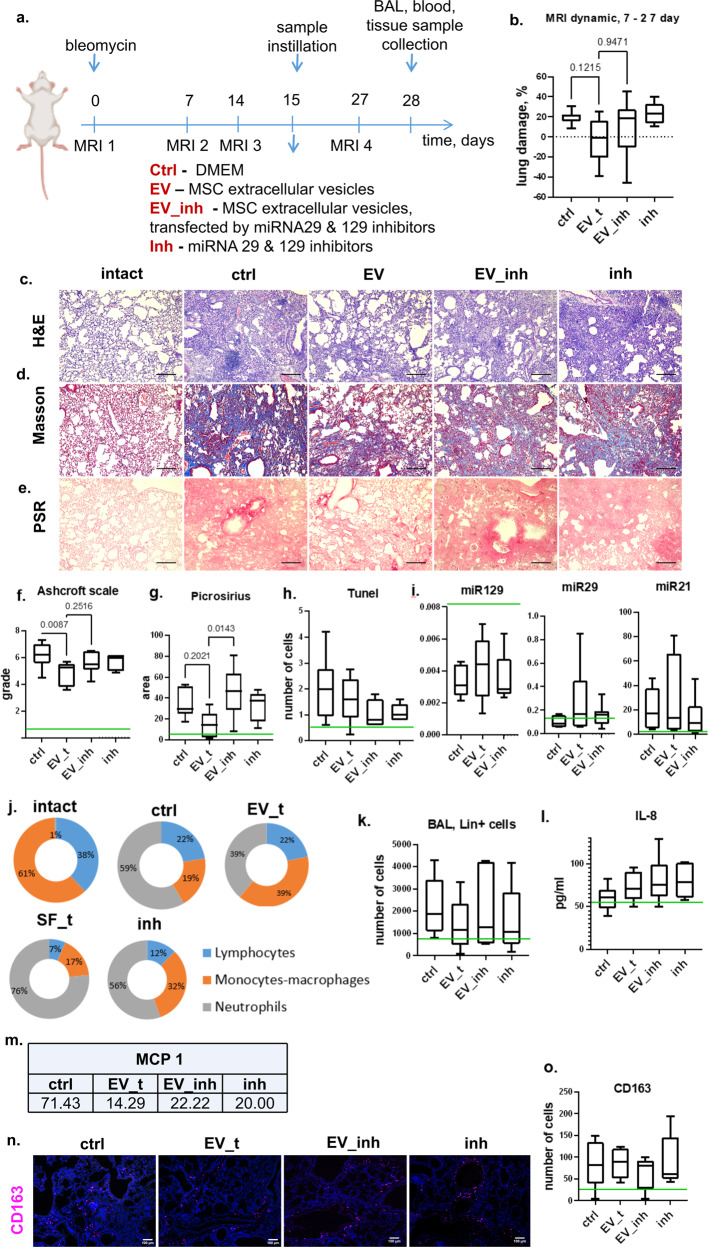
Transfer of microRNA-29c and microRNA-129 mediates the effects of MSC-EVs on pulmonary fibrosis resolution. **a** Schematic design of the treatment of bleomycin-induced fibrosis in C57BL/6 mice; Ctrl (DMEM 14 days after bleomycin administration), *n* = 11; EV_t (MSC extracellular vesicles 14 days after bleomycin administration), *n* = 9; EV_inh (MSC extracellular vesicles transfected by miRNA-29 and miRNA-129 inhibitors 14 days after bleomycin administration), *n* = 9; inh (miRNA-29 and miRNA-129 inhibitors 14 days after bleomycin administration), *n* = 5; *n* = biologically independent animals per group. **b** Quantification of dynamic changes in the lung tissue density measured via MRI. **c**–**e** Representative image of **c** hematoxylin–eosin (H&E), **d** Masson trichrome, and **e** Picrosirius Red (PSR) staining. Scale bar = 100 μm. **f** Quantification of pulmonary fibrosis severity using the Ashcroft scale. The assessment was conducted by two independent blinded experts. **g** Quantification of ECM deposition on the PSR staining image. **h** Quantification of apoptotic cells in the pulmonary tissue measured via TUNEL staining. **i** PCR analysis of the miRNA-129, miRNA-29c, and miRNA-21 levels in the lung tissue. **j** Quantification of immune cell types on the BAL smears. The assessment was conducted by two independent blinded experts. **k** Quantification of the major hematopoietic cell lineages in the BAL fluid via flow cytometry. **l** Quantification of IL-6 in the BAL fluid using ELISAs. **m** Percentage of animals with a detected rate of MCP-1 in the BAL using ELISA. **n** Representative images of pulmonary tissue immunocytochemical analysis for CD163. Scale bar = 100 μm. **o** Quantification of CD163-positive cells in the pulmonary tissue. The green line indicates the median for the intact group.

**Fig. 6:**
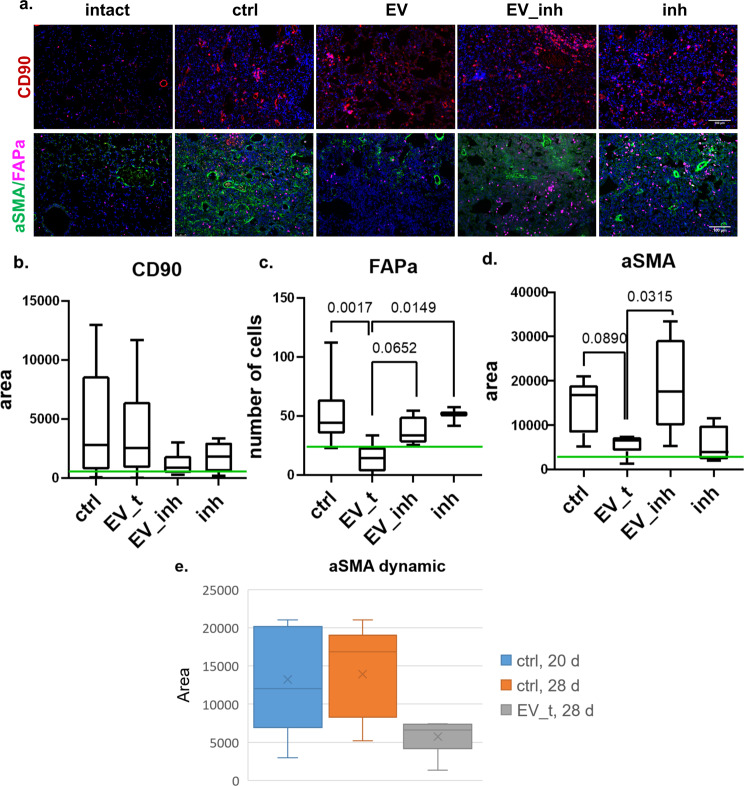
MSC-EVs stimulate the dedifferentiation of myofibroblasts and myofibroblast precursors (FAPa^+^) in fibrotic pulmonary tissue through the transfer of miR-29c and miR-129 in vivo. **a** Representative images of pulmonary tissue immunocytochemical analysis for CD90 (top panel), aSMA (green), and fibroblast activation protein A (FAPa, red) (bottom panel) expression. Scale bar = 100 μm. **b**–**d** Quantification of CD90- (**c**), aSMA- (**d**), and FAPa- (**e**) positive cells measured by average cell area (**c**, **d**) or number of cells (**e**) in pulmonary tissue sections. The green line indicates the median for the intact group. Ctrl (DMEM 14 days after bleomycin administration), *n* = 11; EV_t (MSC extracellular vesicles 14 days after bleomycin administration), *n* = 9; EV_inh (MSC extracellular vesicles transfected by miRNA-29c and miRNA-129 inhibitors 14 days after bleomycin administration), *n* = 9; inh (miRNA-29c and miRNA-129 inhibitors 14 days after bleomycin administration), *n* = 5; *n* = biologically independent animals per group. The number of analyzed fields of view per sample = 5–9. **e** aSMA dynamics.

To evaluate the contribution of miR-29c and miR-129 transfer to the ability of hMSC-EVs to induce fibrosis resolution in vivo, we applied hMSC-EVs transfected by antagomirs to these miRs or antagomirs without EVs 2 weeks after the induction of pulmonary fibrosis in mice (Fig. 5a). The transfection protocol per se did not affect the antifibrotic abilities of hMSC-EVs (Supplementary Fig. 7). The introduction of native hMSC-EVs did not significantly affect the miR-29c and miR-129 contents in the lung tissue (Fig. 5i). However, hMSC-EV transfection by antagomirs as well as antagomirs themselves abolished the ability of hMSC-EVs to attenuate fibrosis progression and induce fibrosis resolution. Thus, the area of fibrotic damage evaluated *via* MRI did not decrease after the administration of transfected hMSC-EVs in contrast to native EVs (Fig. 5b, Supplementary Fig. 8). Consistently, the morphological analysis revealed more severe fibrosis after the administration of transfected hMSC-EVs: poorer alveolar density and higher Ashcroft score compared with that of native hMSC-EVs (Fig. 5c, d, f). Furthermore, the deposition of collagen, a target of miR-29c and miR-129, was more prominent after the administration of antagomir-transfected hMSC-EVs (Fig. 5e, g). The greatest collagen depositions were observed in the perialveolar and peripleural zones. The numbers of aSMA^+^ and FAPa^+^ cells after the administration of transfected hMSC-EVs significantly increased compared with those of the animals receiving native hMSC-EVs (Fig. 6a–e). The transfection of hMSC-EVs with antagomir-29c and antagomir-129 or antagomirs alone led to a decrease in the macrophage/neutrophil ratio in the BAL fluid and an increase in the concentration of MCP-1 (Fig. 5j, k, m). However, the total number of lin^+^ cells in the BAL fluid or the number of CD163^+^ macrophages in the lungs remained largely unaffected (Fig. 5k, l, n, o).

Taken together, these data indicate that miR-29 and miR-129 are important for hMSC-EVs to facilitate fibrosis resolution, possibly by targeting myofibroblasts and their progenitors, affecting their phenotype and ECM synthesis.

## Discussion

The beneficial effect of MSCs for fibrosis treatment is largely attributed to their secretome and to EVs in particular^19–23^. Our previous in vitro results indicated that hMSC-EVs and hMSC-SFs, added simultaneously with the profibrotic factor TGFβ, prevented fibroblast-to-myofibroblast differentiation^8^. However, contrary to our expectation, the administration of neither hMSC-EV nor hMSC-SFs exerted any protective effect on fibrosis progression in vivo when applied shortly after bleomycin-induced injury. These data contradict the results of Mansouri’s work, which demonstrated that simultaneous administration of hMSC-EVs with bleomycin caused a significant decrease in fibrosis hallmarks, including collagen deposition and the number of fibrotic foci, as well as a switch of monocytes toward the nonactivated, nonclassical CD45^+^/CD11b^+^/MHCII^–^/CD64^–^/CCR-2^–^/Ly6C^l^° phenotype^22^. Such a discrepancy clearly indicates that a precise “therapeutic window” is required for the administration of hMSC-EVs.

Thus, hMSC-EVs but not hMSC-SFs evidently diminished fibrotic signs when administered to mice with pulmonary fibrosis that developed 2 weeks after bleomycin-induced injury. hMSC-EV administration did not affect the number of CD163^+^ macrophages in the lung stroma 4 weeks after bleomycin-induced injury. At this stage, CD163^+^ macrophages facilitate the removal of deposited ECM and are, therefore, necessary for fibrosis resolution^24,25^. Because these macrophages predominantly arise from circulating monocytes^26^, the observed shift in the leukocyte ratio in the BAL fluid toward monocyte-like cells suggests that the antifibrotic effect of hMSC-EVs is partially mediated by enhanced collagen removal.

However, we demonstrated that hMSC-EVs attenuated the accumulation of myofibroblasts, representing a key cell type responsible for excessive ECM production and remodeling. Furthermore, hMSC-EVs suppress the main source of myofibroblasts, namely, activated FAPa^+^ progenitor cells, which could be of multiple origins, including fibroblasts and fibrocytes^27,28^. To the best of our knowledge, this is the first study demonstrating that hMSC-EVs can control the number of FAPa^+^ cells during fibrotic remodeling. FAPa is a prolyl-specific serine protease known as a marker of activated invasive fibroblasts associated with malignant tumors or healing wounds^29,30^. The contribution of FAPa^+^ cells to fibrosis development and fibrotic focus remodeling is considered ambiguous^31–33^. FAPa^+^, as invasive cells, can be responsible for fibrosis expansion, increasing the area of the compromised tissue. However, due to proteolytic activity, these cells can promote matrix remodeling and fibrotic foci disassembly^34^. Here, we also demonstrated that the accumulation of FAPa^+^ cells within lung tissue correlates with the severity of bleomycin-induced fibrosis. Moreover, these cells reside predominantly along the periphery but outside the fibrotic foci, whereas double-positive FAPa^+^/aSMA^+^ cells are located at the very border of the focus. These findings indicate that FAPa^+^ cells are likely to be attracted toward the fibrotic foci from the lung interstitium and contribute to its expansion by a subsequent transformation into FAPa^−^/aSMA^+^ myofibroblasts.

According to recent studies, miR transfer by MSC-EVs essentially contributes to their antifibrotic activity targeting multiple cell types within the lung tissue^2^. Our data are consistent with these observations, as we demonstrate that RNase treatment impairs the ability of hMSC-EVs to facilitate fibrosis resolution. Notably, in different models of fibrosis, the effects of various cargoes within MSC-EVs were observed, including both miRs and mRNAs, which mediated the beneficial effects in the resolution of fibrosis and prevention of its progression^23^. Importantly, such abundant evidence makes MSC-EVs an attractive tool in the development of antifibrotic therapies. Apparently, MSC-EVs can be a multitarget drug with a pleiotropic mode of action that has a beneficial effect on several key profibrogenic pathways at once.

Specifically, we evaluated the contribution of selected miRs considered fibrosis-related via bioinformatic analysis to the antifibrotic effects of hMSC-EVs. We demonstrated that the inhibition of miR-21 within hMSC-EVs promotes their antifibrotic properties. These data correlate with the well-established ability of miRNA-21 within stromal cell exosomes to regulate stromal cell synthetic and contraction activities^35,36^ and maintain a profibrotic phenotype of MSCs themselves^37^. In contrast, we elegantly demonstrated here that both miR-29c and miR-129 inhibition significantly attenuated the ability of hMSC-EVs to facilitate fibrosis resolution in vivo. Because myofibroblasts are the principal source of ECM overproduction, the widely considered approach for their elimination is fibrosis treatment. Several approaches aimed at myofibroblast elimination have been proposed thus far, including the activation of their apoptosis, prevention of their differentiation, and induction of myofibroblast dedifferentiation^27,38–40^.

Because the elimination of myofibroblasts, which are resistant to apoptosis, is important for fibrosis resolution^27^, we examined the potential effect of miR-29c and miR-129 on their susceptibility to apoptosis. Thus, miR-29c was shown to inhibit excessive autophagy via the PTEN/Akt/mTOR signaling pathway^35,41^. Furthermore, miR-29c and miR-129 have common targets that are involved in the regulation of apoptosis, including XIAP and MTHFD2^27,42^. Despite these potential mechanisms, we did not observe a significant effect of hMSC-EVs on apoptotic cell death in the lung tissue.

Another promising approach for fibrosis treatment is the management of the differentiation of myofibroblasts and their precursors. Several studies have demonstrated that MSCs are able to control the differentiation of various cell types^19,43,44^, including myofibroblast precursors^8,45–47^. Differentiated myofibroblasts are another potential target of hMSC-EVs. The role of dedifferentiation in removing myofibroblasts in vivo, at least for some subpopulations of myofibroblasts, was actively discussed in recent studies^39,48^.

Although miR-29c and miR-129 do not directly target FAPα or αSMA, there are several ways in which these miRs could be involved in the regulation of myofibroblast dedifferentiation. Thus, miR-29c suppresses the WNT/b-catenin signaling pathway^44^, and miR-129 participates in epithelial cell activation during EMT^47^, potentially facilitating FAPa^+^ cell accumulation. Moreover, the Rel-subunit of the NFkB complex is a direct target for miR-29c, whereas the activation of the NFkB signaling pathway is correlated with an increase in FAPα expression^49^. In humans, miR-29c and miR-129 could also potentially target SMAD2, SMAD4, and SP1, the most critical regulators of αSMA and FAPα expression. Both miRs regulate the expression of multiple ECM proteins involved in fibrotic processes^15,50^, including collagen type I^51^, the most important factor supporting myofibroblast differentiation^28,52^. Notably, our data confirmed that the transfer of miR-29c and miR-129 within hMSC-EVs into myofibroblasts significantly affects collagen type I expression in target cells. We also showed that inhibition of these miRs within hMSC-EVs can increase the number of proteins included in the focal adhesion complex: alpha-actinins and vinculin. Alpha-actinins are a family of proteins involved in the formation of the three-dimensional organization of actin fibrils and focal adhesions^53,54^. An increase in alpha-actinin expression during fibroblast-to-myofibroblast differentiation was recently demonstrated^55^. The role of vinculin in the maintenance of the myofibroblast phenotype is well established^56–58^. We assume that a decrease in the amount of these proteins and the associated disassembly of focal adhesions can lead to a change in the mechanosensitivity of myofibroblasts and, as a result, disassembly of actin filaments and myofibroblast dedifferentiation.

Importantly, observing the labeled hMSC-EV biodistribution in mice after intratracheal administration, we demonstrated that their retention within the lung tissue was rather short, regardless of whether the beneficial effects were realized during at least 2 weeks, which supported the hypothesis of profibrotic cell reprogramming as a key mechanism of hMSC-EV antifibrotic activity. Taken together, our data suggest that hMSC-EVs facilitate fibrosis resolution by both suppressing the differentiation of FAPa^+^ cells and inducing myofibroblast dedifferentiation. In the lungs, MSCs are predominantly located in the perivascular or perialveolar compartments^59^, which makes their contact with myofibroblasts and their precursors, which are also located in these niches, possible.

We acknowledge several limitations to our study. First, it is still unclear whether the revealed mechanisms of the MSC secretome antifibrotic effect are universal or specific to pulmonary fibrosis. Second, MSC secretome components were administered once in our proof-of-concept study, so a further dose–response investigation is absolutely necessary to determine the optimal dosage regimen and duration of treatment by hMSC-EVs for modulating fibrosis. Third, several manufacturing-related issues should be addressed to develop a prototype of hMSC-EV-based antifibrotic drugs for translational studies. Finally, it would be clinically and pharmaceutically beneficial to directly compare hMSC-EVs and MSCs or hMSC-EVs and selected miRs in terms of their capability to achieve therapeutic effects. Additionally, other functional components of the MSC secretome could be involved in the ability of MSCs to attenuate fibrosis progression and induce fibrosis resolution, which warrants further research.

## Supplementary information

SUPPLEMENTAL MATERIAL
