# Supplementary material for: Mesenchymal stromal cells facilitate resolution of pulmonary fibrosis by miR-29c and miR-129 intercellular transfer
Source: Exp Mol Med. 2023 Jul 3;55(7):1399–412. doi: 10.1038/s12276-023-01017-w (PMC10393964; doi:10.1038/s12276-023-01017-w)
Supplement: Supplementary file 1 — SUPPLEMENTAL MATERIAL [file 12276_2023_1017_MOESM1_ESM.pdf]

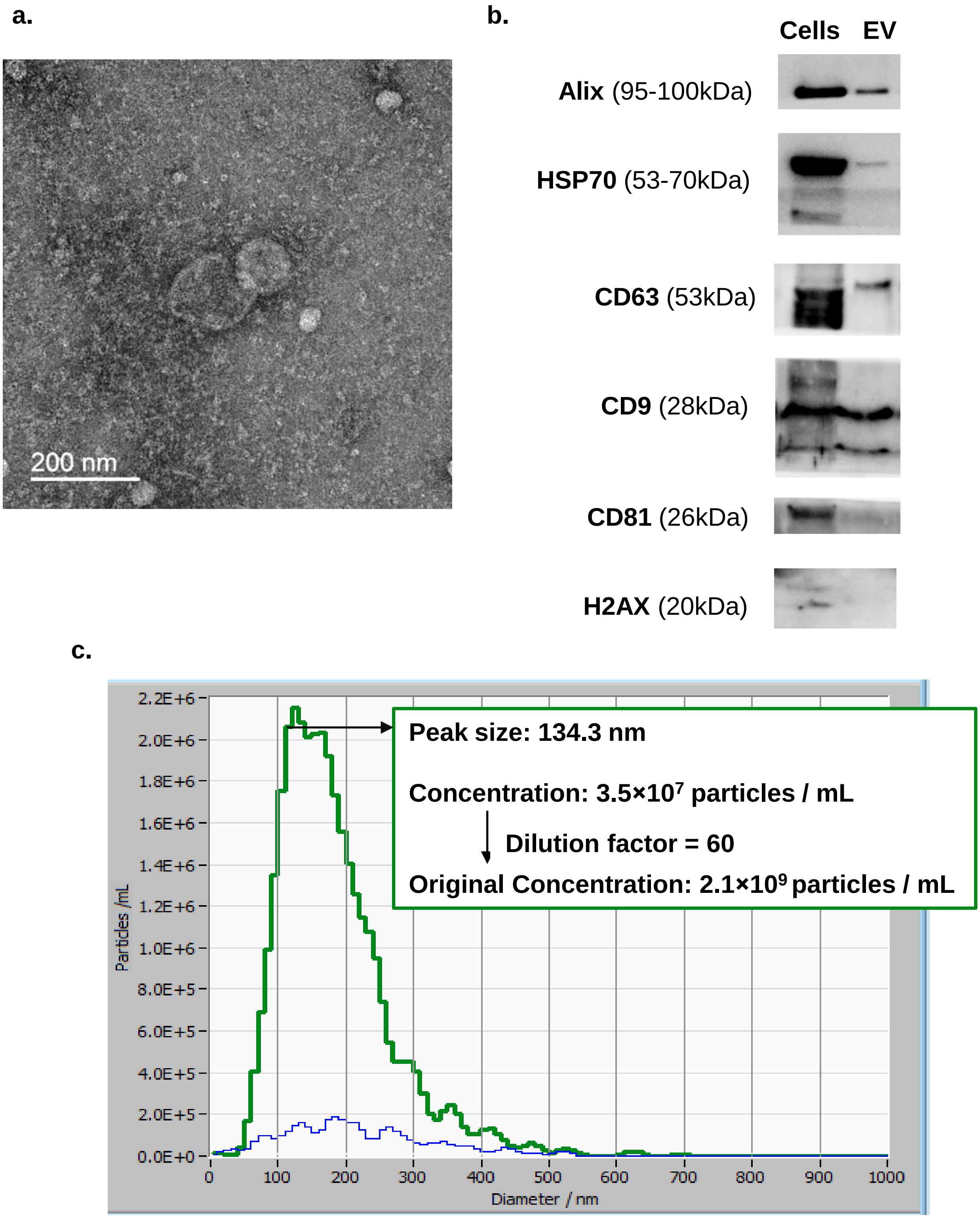

Supplementary Fig. 1. MSC-EV characteristics.

a. Representative image of hMSC-EV visualized *via* transmission electron microscopy (TEM). Scale bar = 200 nm. **b.** Immunoblotting for Alix, HSP70, CD63, CD9, CD81, and H2AX in hMSC-EV and MSC lysates. **c.** Size and concentration analysis of non-concentrated hMSC-EV after 2 days of conditioning (green) and control aliquots of MSC-CM after 30 min of conditioning (blue) by NTA using ZetaView.

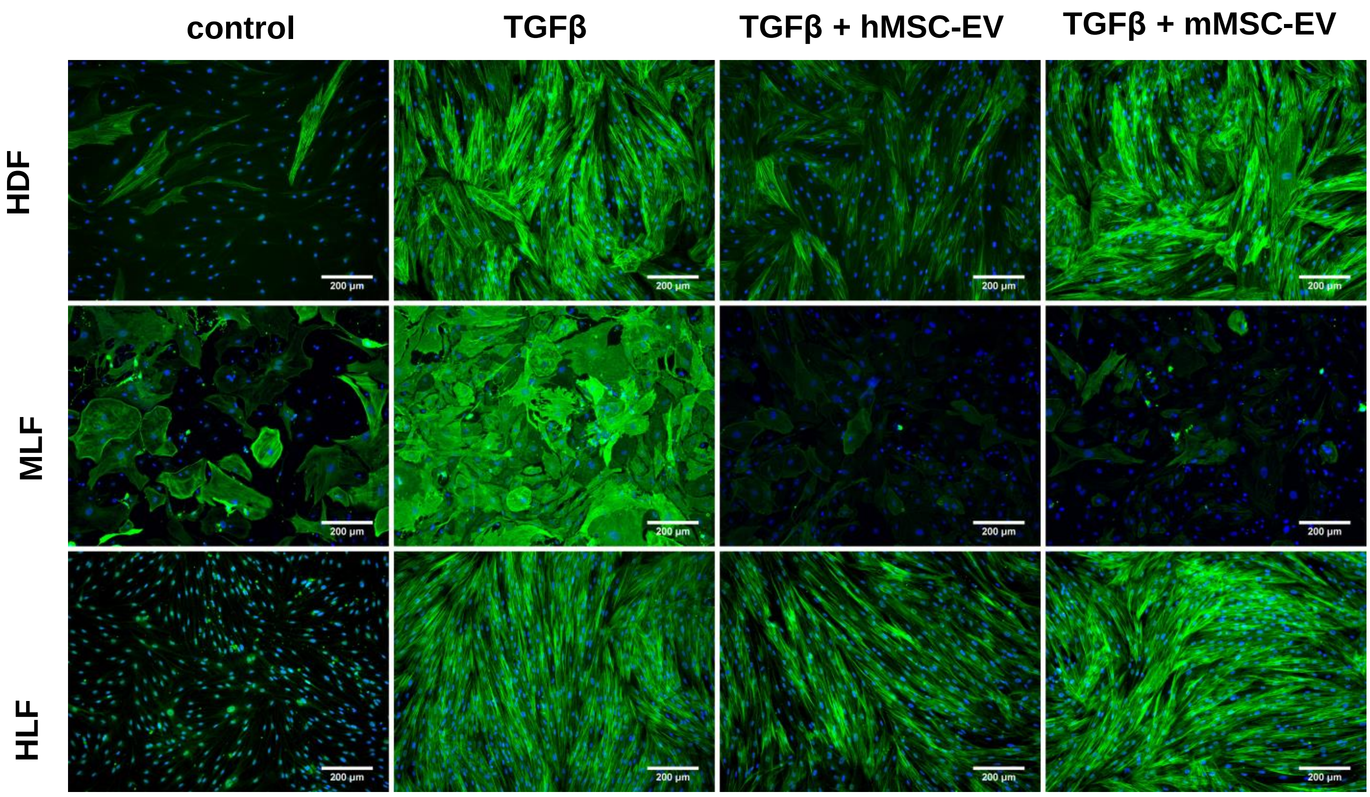

Supplementary Fig. 2. Comparison of murine and human MSC-EV antifibrotic effects *in vitro*. Representative images of HDF, MLF, and HLF immunocytochemical analysis for the αSMA expression. The cells were incubated with TGFb for 4 days (TGFb) in the presence of human MSC-EV (TGFb + hMSC-EV) or murine MSC-EV (TGFb + mMSC-EV) and compared with the cells cultured without TGFb (control). Scale bar = 200 µm.

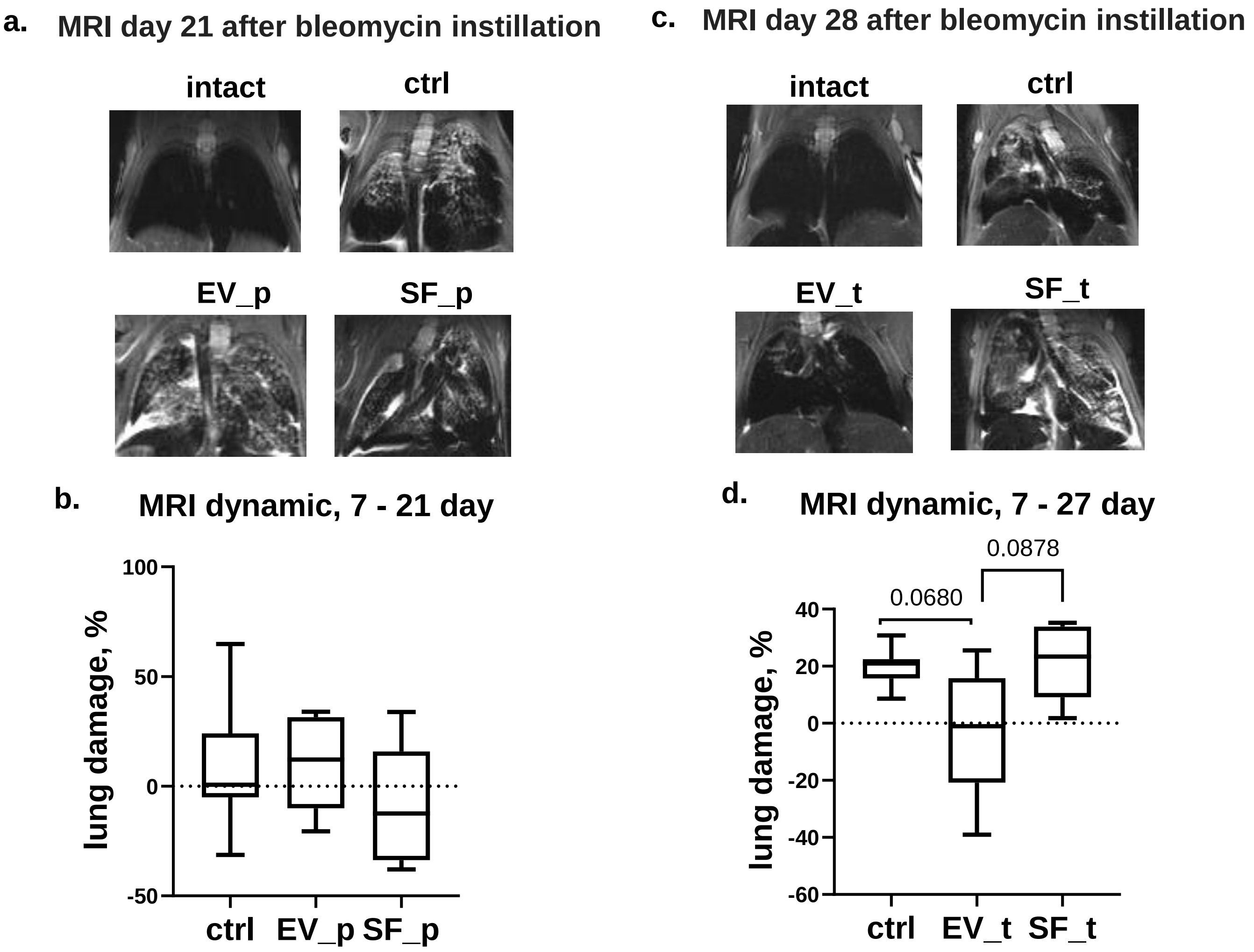

Supplementary Fig. 3. Dynamic changes in the lung tissue density in mice with bleomycin-induced pulmonary fibrosis treated with MSC-EV measured *via* MRI. a. Representative MR images for the fibrosis prevention groups. **b.** Quantification of dynamic changes in the lung tissue density measured *via* MRI in **the** fibrosis prevention groups. **a–b Ctrl** (DMEM 1 day after bleomycin administration), n = 11; **EV_p** (MSC extracellular vesicles 1 day after bleomycin administration), n = 9; **SF_p** (MSC soluble factors 1 day after bleomycin administration), n = 6, n = biological independent animals per group. Number of analyzed fields of view per sample = 3–10 for each animal. **c.** Representative MR image for the fibrosis treatment groups. **d.** Quantification of dynamic changes in the lung tissue density measured *via* MRI in the fibrosis treatment groups. **c–d**; **Ctrl** (DMEM 14 days after bleomycin administration), n = 11; **EV_t** (MSC extracellular vesicles 14 days after bleomycin administration), n = 9; **SF_t** (MSC soluble factors 14 days after bleomycin administration), n = 6; n = biological independent animals per group. Number of analyzed fields of view per sample = 3–10 for each animal.

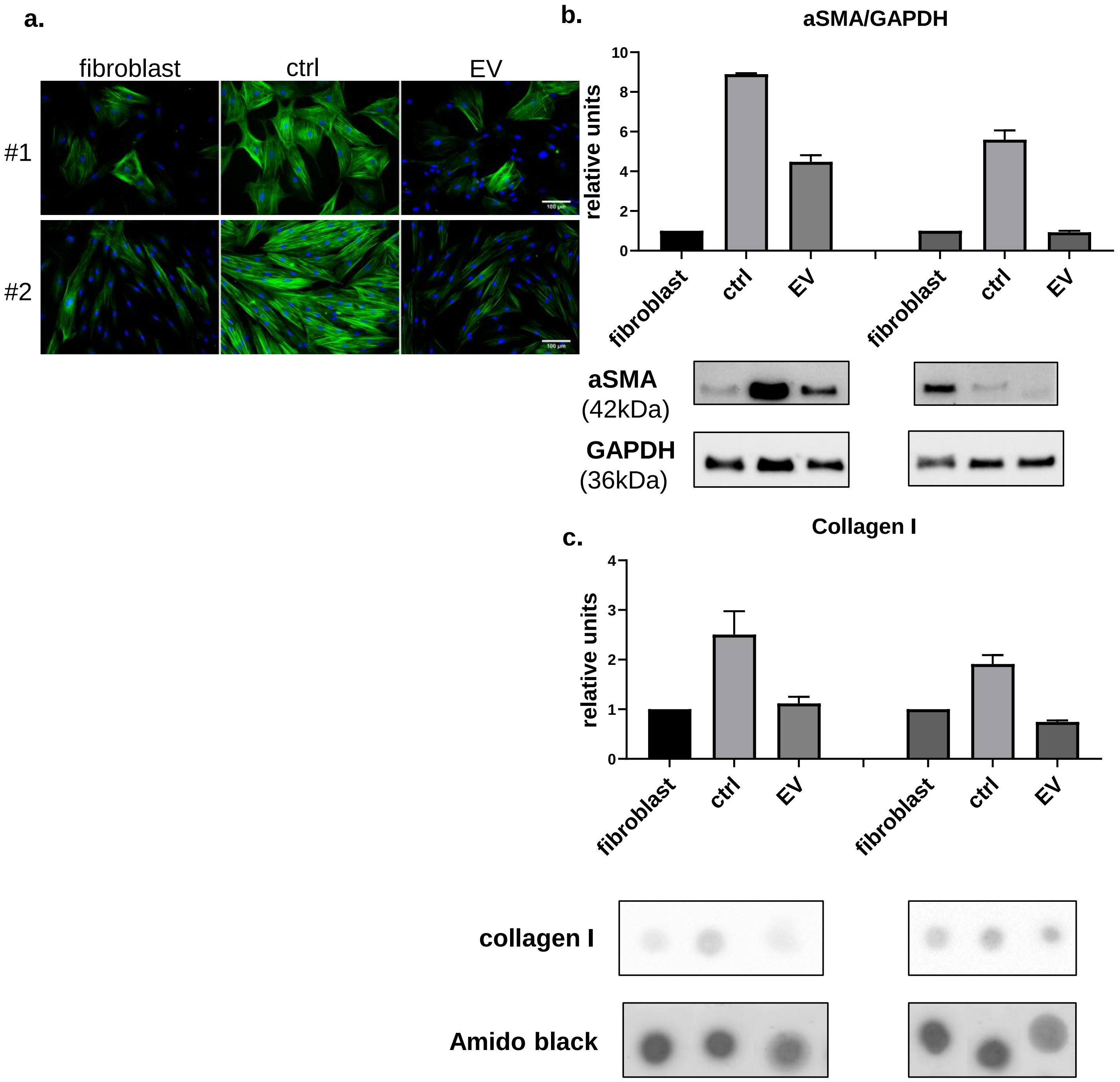

Supplementary Fig. 4. Isolation of MSC-EV by gradient ultracentrifugation does not change their antifibrotic effect.

a. Representative image of myofibroblast immunocytochemical analysis for the αSMA expression. Scale bar = 100 μm. **b**. Western-blot analysis of aSMA. *n* = 2 biological independent experiments. **c.** Dot blot analysis of collagen type I. *n* = 2 biological independent experiments for all methods. #1 and #2 – independent data for two different HDF donors. **Fibroblast** – undifferentiated fibroblasts, **ctrl** – myofibroblasts treated with DMEM during 4 days, **EV** - myofibroblasts treated with EV during 4 days.

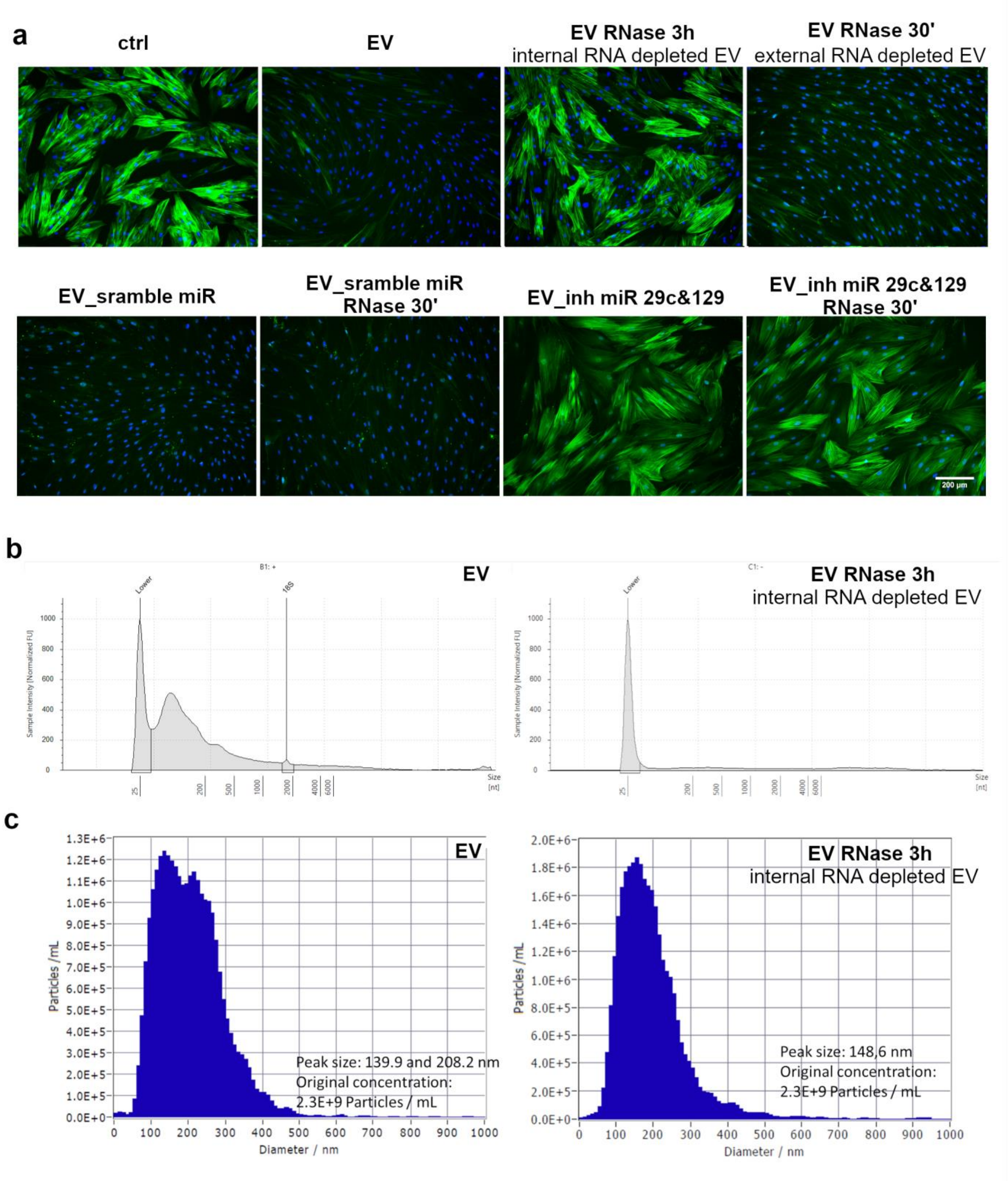

Supplementary Fig. 5. Critical contribution of RNAs to the MSC-EV antifibrotic effects, part 1. a. Representative image of the myofibroblast immunocytochemical analysis for the αSMA expression in the presence of MSC-EV treated with RNase. MSC-EV treated with RNAse for 3 h (EV RNase 3h, internal RNA-depleted EV) or for 30 min (EV RNase 30’, external RNA-depleted EV), MSC-EV transfected by scramble miR ± RNase 30-min treatment, MSC-EV transfected by the miR-29c and miR129 inhibitor ± RNase 30-min treatment. n = 3 biological independent experiments. b. Analysis of the length distribution of nucleic acid fragments within native MSC-EV or MSC-EV treated by RNAse for 3 h. c NTA for MSC-EV or MSC-EV treated by RNAse for 3 h.

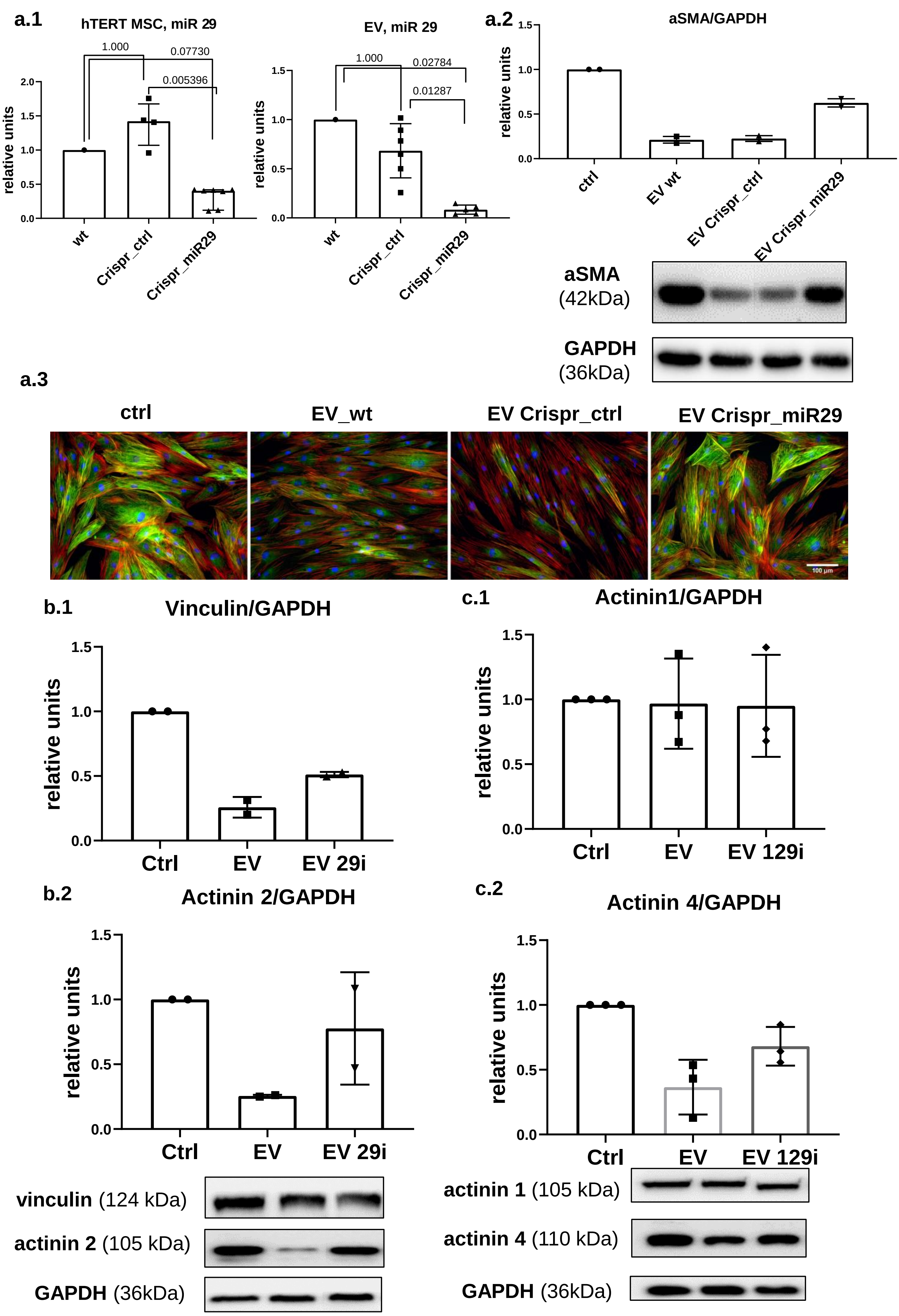

Supplementary Fig. 6. Critical contribution of RNAs to the MSC-EV antifibrotic effects, part 2. a. Representative image of the myofibroblast immunocytochemical analysis for the αSMA expression in the presence of EV from MSC with Crispr-Cas9 miR-29 knockdown. Wt, EV from hTERT-MSC ; Crispr_ctrl, EV from hTERT-MSC with Crispr-Cas9 nonsense knockdown; and Crispr_miR29, EV from hTERT-MSC with Crispr-Cas9 miR-29 knockdown. b.1 miR29 expression within hTERT-MSC (left graph) or appropriate EV (right graph), RT-PCR. b.2,3. Analysis of the aSMA expression within the myofibroblasts after exposure to serum-free cultured control (ctrl) or hTERT-MSC EV (EV wt), hTERT-MSC Crispr_ctrl EV (EV Crispr_ctrl), or hTERT-MSC Crispr_miR29 EV (EV Crispr_miR29). b.2 Western blotting, b.3 immunocytochemical analysis, n = 2. b,c Western blot analysis specific targets for miR-29c (b1, b2. n = 2 biological independent experiments) and miR-129 (c1, c2. n = 3 biological independent experiments)

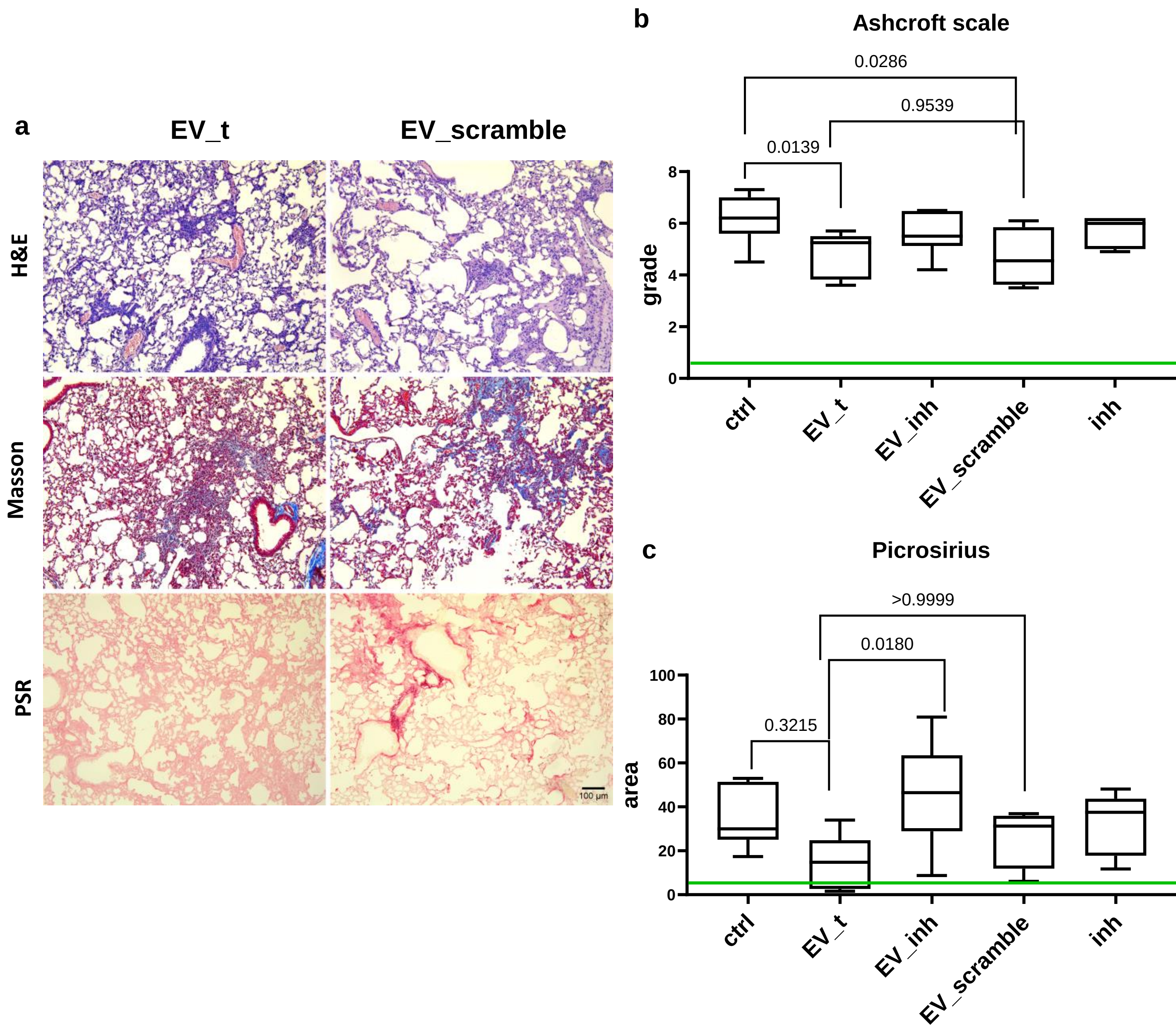

Supplementary Fig. 7. **MSC-EV transfection with scramble oligos as controls for specific miR inhibitors does not affect MSC-EV antifibrotic effects *in vivo*. a.** Representative images of hematoxylin–eosin (H&E), Masson trichrome and Picrosirius Red (PSR) staining. Scale bar = 100 µm. **b.** Quantification of the pulmonary fibrosis severity using the Ashcroft scale; **Ctrl** (DMEM 14 days after bleomycin administration), n = 11; **EV_t** (MSC extracellular vesicles 14 days after bleomycin administration), n = 9; **EV_inh** (MSC extracellular vesicle, transfected by the miRNA-29 & miRNA-129 inhibitors 14s day after bleomycin administration), n = 8; **inh** (miRNA-29 and miRNA-129 inhibitors 14 days after bleomycin administration), n = 5; **EV_scramble** (MSC extracellular vesicle, transfected by the miRNA Inhibitor Control 14s day after bleomycin administration), n= 4, n = biological independent animals per group. Assessment was conducted by two independent blinded experts. **c.** Quantification of the ECM deposition on the PSR staining image. The green line indicates the median for the intact group.

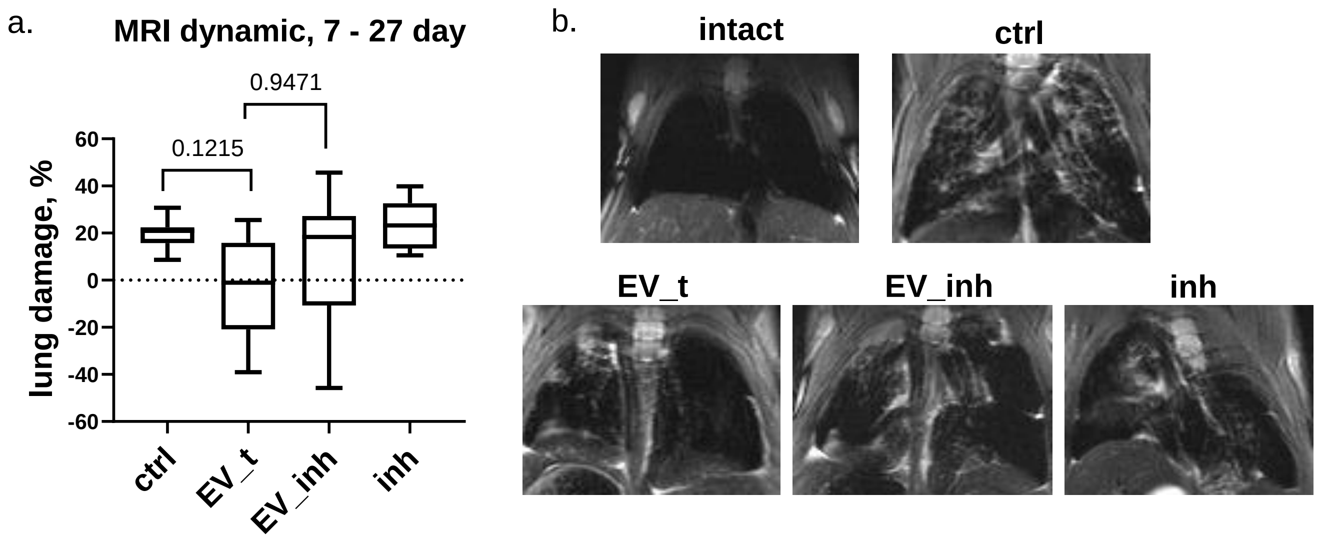

Supplementary Fig. 8. **Dynamic changes in the lung tissue density in mice with bleomycin-induced pulmonary fibrosis treated with MSC-EV transfected by miR inhibitors measured *via* MRI.** a. Quantification of dynamic changes in the lung tissue density measured *via* MRI. b. Representative MR images. **Ctrl** (DMEM 14 days after bleomycin administration), n = 11; **EV_t** (MSC extracellular vesicles 14 days after bleomycin administration), n = 9; **EV_inh** (MSC extracellular vesicle, transfected by the miRNA-29 & miRNA-129 inhibitors 14s day after bleomycin administration), n = 9; **inh** (miRNA-29 and miRNA-129 inhibitors 14 days after bleomycin administration), n = 5; n = biological independent animals per group. Number of analyzed fields of view per sample = 3–10 for each animal.

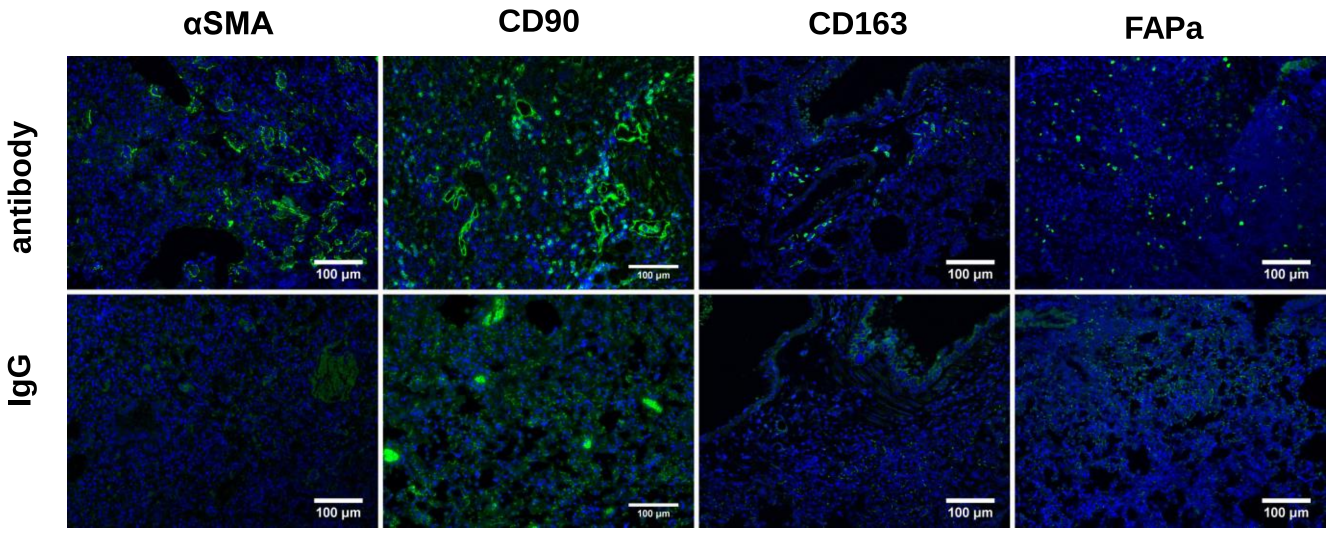

Supplementary Fig. 9. Immunohistochemical analysis for the aSMA, CD90, CD163, and FAPa expressions in pulmonary tissue (antibody) compared with staining with the corresponding isotype control antibodies (IgG). Scale bar = 100 μm.

MicroRNA and Target Gene Description:

**miRNA Name**	hsa-miR-129-5p	**miRNA Sequence**	CUUUUUGCGGUCUGGGCUUGC
**Previous Name**	hsa-miR-129		
**Target Score**	90	**Seed Location**	96, 225
**NCBI Gene ID**	1277	**GenBank Accession**	NM_000088
**Gene Symbol**	COL1A1	**3' UTR Length**	1406
**Gene Description**	collagen type I alpha 1 chain		

3' UTR Sequence

```
   1 actccctcca tcccaacctg gctccctccc acccaaccaa ctttcccccc aacccggaaa
  61 cagacaagca acccaaactg aaccccctca aaagccaaaa aatgggagac aatttcacat
 121 ggactttgga aaatattttt ttcctttgca ttcatctctc aaacttagtt tttatctttg
 181 accaaccgaa catgaccaaa aaccaaaagt gcattcaacc ttaccaaaaa aaaaaaaaaa
 241 aaaagaataa ataaataact ttttaaaaaa ggaagcttgg tccacttgct tgaagaccca
 301 tgcgggggta agtccctttc tgcccgttgg gcttatgaaa ccccaatgct gccctttctg
 361 ctcctttctc cacacccccc ttggggcctc ccctccactc cttcccaaat ctgtctcccc
 421 agaagacaca ggaaacaatg tattgtctgc ccagcaatca aaggcaatgc tcaaacaccc
 481 aagtggcccc caccctcagc ccgctcctgc ccgcccagca cccccaggcc ctggggggacc
 541 tggggttctc agactgccaa agaagccttg ccatctggcg ctcccatggc tcttgcaaca
 601 tctccccttc gtttttgagg gggtcatgcc gggggagcca ccagcccctc actgggttcg
 661 gaggagagtc aggaagggcc acgacaaagc agaaacatcg gatttgggga acgcgtgtca
 721 atcccttgtg ccgcagggct gggcgggaga gactgttctg ttccttgtgt aactgtgttg
 781 ctgaaagact acctcgttct tgtcttgatg tgtcaccggg gcaactgcct gggggcgggg
 841 atgggggcag ggtggaagcg gctccccatt ttataccaaa ggtgctacat ctatgtgatg
 901 ggtggggtgg ggagggaatc actggtgcta tagaaattga gatgcccccc caggccagca
 961 aatgttcctt tttgttcaaa gtctattttt attccttgat atttttcttt tttttttttt
1021 tttttgtgg atggggactt gtgaattttt ctaaaggtgc tatttaacat gggaggagag
1081 cgtgtgcggc tccagcccag cccgctgctc actttccacc ctctctccac ctgcctctgg
1141 cttctcaggc ctctgctctc cgacctctct cctctgaaac cctcctccac agctgcagcc
1201 catcctcccg gctccctcct agtctgtcct gcgtcctctg tccccgggtt tcagagacaa
1261 cttcccaaag cacaaagcag tttttccccc taggggtggg aggaagcaaa agactctgta
1321 cctattttgt atgtgtataa taatttgaga tgtttttaat tattttgatt gctggaataa
1381 agcatgtgga aatgacccaa acataa
```

**miRNA Name**	mmu-miR-129-5p	**miRNA Sequence**	CUUUUUGCGGUCUGGGCUUGC
**Previous Name**	mmu-miR-129		
**Target Score**	60	**Seed Location**	228, 1421
**NCBI Gene ID**	12842	**GenBank Accession**	NM_007742
**Gene Symbol**	Col1a1	**3' UTR Length**	1439
**Gene Description**	collagen, type I, alpha 1		

3' UTR Sequence

```
   1 actccctcca ccccaatctg gttccctccc acccagccca cttttcccca accctggaaa
  61 cagacgaaca acccaaactc aatttccccc aaaagccaaa aatatgggag ataatttcac
 121 atggactttg gaaaacattt tttttccttt gcattcacct ttcaaactta gtttttacct
 181 ttgaccaact gaacgtgacc aaaaaccaaa agtgcattca accttaccaa aaaagaaaaa
 241 aaaaaaaaga ataaataaat aactttttaa aaaaggaagc ttggtcctct tgcttgaaga
 301 cctatgtggg tataagtccc ttcctgccca cttggcttat gataccctaa tgctgccttt
 361 tctgctcctt tctccacccc ctcttggggc ctctcctcca ttgctcccca aatttaagtc
 421 tcccccaaga cacaggaaat aatgcattgt ctgcccagca aacaaaggca atgctgaaat
 481 gtcccaccag cccctcaacc ccgtctactt ccctacccag caccctcaaa tcctgctggg
 541 acatggggtt cttggactgt tgaaggaacc taaccatctg gcatctccat ggcctctgca
 601 acaaaccccc cacttttttc tctccccccc cccccaggga gggcctgtgc tttgggcagc
 661 cacctgcccc tctcaggggt ttggagccag gcagggtcac agcagactgg aaacatcgga
 721 catgcatgtg caggctgggt gggagagacc gttctattcc tcagtgcaat tgtgttgctg
 781 aaagactacc tcgttcttgt ctttgtgtgt caccggggca actgtgtggg ggcggggatg
 841 ggggcagggt ggcagcacgc ccagtttggt atcaaaggtg ctacatctct gtgaaggggt
 901 ggggtgggaa ggaatttctg gtgctataga agctgagatg ctccctagac cagcaaatgt
 961 ttcttttgtt caaagtattt tttattcttt tttttttttt ttttaatgga tagggacttg
1021 tgtgaattgt tggggttttt tttttttttt tttttggttt tgtttttttt tgttttgttt
1081 tgtttttttt cctgaaggtg ctatttaaca agggagaaga gagtgcgggg acttcaccct
1141 gcccactctc tactctctct ccactcttct agttcctggg cctatctgat ctctctcttt
1201 cttctgaaac cctcccctct tgctgctgct ccctcccctc tgcctctctc ttggtctgtc
1261 ctgcatcagg gtttcagagc accactttcc aaagcacaaa acagttttta cccctgggct
1321 gggaggaaac aagagactct gtacctattt tgtatgtgta taataatttg agatgttttt
1381 aattattttg attgctggaa taaagcatgt ggaaatgacc caaaaaaaaa aaaaaaaaa
```

Supplementary Table 1. hsa-microRNA-129-5p and mmu-microRNA-129-5p have binding sites on collagen type I alpha 1

MicroRNA and Target Gene Description:

**miRNA Name**	hsa-miR-29c-3p	**miRNA Sequence**	UAGCACCAUUUGAAAUCGGUUA
**Previous Name**	hsa-miR-29c		
**Target Score**	98	**Seed Location**	881, 923, 1056
**NCBI Gene ID**	1277	**GenBank Accession**	NM_000088
**Gene Symbol**	COL1A1	**3' UTR Length**	1406
**Gene Description**	collagen type I alpha 1 chain		

3' UTR Sequence

```
   1 actccctcca tcccaacctg gctccctccc acccaaccaa ctttcccccc aacccggaaa
  61 cagacaagca acccaaactg aaccccctca aaagccaaaa aatgggagac aatttcacat
 121 ggactttgga aaatattttt ttcctttgca ttcatctctc aaacttagtt tttatctttg
 181 accaaccgaa catgaccaaa aaccaaaagt gcattcaacc ttaccaaaaa aaaaaaaaaa
 241 aaaagaataa ataaataact ttttaaaaaa ggaagcttgg tccacttgct tgaagaccca
 301 tgcgggggta agtccctttc tgcccgttgg gcttatgaaa ccccaatgct gccctttctg
 361 ctcctttctc cacacccccc ttggggcctc ccctccactc cttcccaaat ctgtctcccc
 421 agaagacaca ggaaacaatg tattgtctgc ccagcaatca aaggcaatgc tcaaacaccc
 481 aagtggcccc caccctcagc ccgctcctgc ccgcccagca cccccaggcc ctgggggacc
 541 tggggttctc agactgccaa agaagccttg ccatctggcg ctcccatggc tcttgcaaca
 601 tctccccttc gtttttgagg gggtcatgcc gggggagcca ccagcccctc actgggttcg
 661 gaggagagtc aggaagggcc acgacaaagc agaaacatcg gatttgggga acgcgtgtca
 721 atcccttgtg ccgcagggct gggcgggaga gactgttctg ttccttgtgt aactgtgttg
 781 ctgaaagact acctcgttct tgtcttgatg tgtcaccggg gcaactgcct gggggcgggg
 841 atgggggcag ggtggaagcg gctccccatt ttataccaaa ggtgctacat ctatgtgatg
 901 ggtggggtgg ggagggaatc actggtgcta tagaaattga gatgcccccc caggccagca
 961 aatgttcctt tttgttcaaa gtctattttt attccttgat atttttcttt tttttttttt
1021 ttttttgtgg atggggactt gtgaattttt ctaaaggtgc tatttaacat gggaggagag
1081 cgtgtgcggc tccagcccag cccgctgctc actttccacc ctctctccac ctgcctctgg
1141 cttctcaggc ctctgctctc cgacctctct cctctgaaac cctcctccac agctgcagcc
1201 catcctcccg gctccctcct agtctgtcct gcgtcctctg tccccgggtt tcagagacaa
1261 cttcccaaag cacaaagcag tttttccccc taggggtggg aggaagcaaa agactctgta
1321 cctattttgt atgtgtataa taatttgaga tgtttttaat tattttgatt gctggaataa
1381 agcatgtgga aatgacccaa acataa
```

MicroRNA and Target Gene Description:

**miRNA Name**	mmu-miR-29c-3p	**miRNA Sequence**	UAGCACCAUUUGAAAUCGGUUA
**Previous Name**	mmu-miR-29c		
**Target Score**	95	**Seed Location**	877, 919, 1097
**NCBI Gene ID**	12842	**GenBank Accession**	NM_007742
**Gene Symbol**	Col1a1	**3' UTR Length**	1439
**Gene Description**	collagen, type I, alpha 1		

3' UTR Sequence

```
   1 actccctcca ccccaatctg gttccctccc acccagccca cttttcccca accctggaaa
  61 cagacgaaca acccaaactc aatttccccc aaaagccaaa aatatgggag ataatttcac
 121 atggactttg gaaaacattt tttttccttt gcattcacct ttcaaactta gtttttacct
 181 ttgaccaact gaacgtgacc aaaaaccaaa agtgcattca accttaccaa aaaagaaaaa
 241 aaaaaaaaga ataaataaat aactttttaa aaaaggaagc ttggtcctct tgcttgaaga
 301 cctatgtggg tataagtccc ttcctgccca cttggcttat gataccctaa tgctgccttt
 361 tctgctcctt tctccacccc ctcttggggc ctctcctcca ttgctcccca aatttaagtc
 421 tcccccaaga cacaggaaat aatgcattgt ctgcccagca aacaaaggca atgctgaaat
 481 gtcccaccag cccctcaacc ccgtctactt ccctacccag caccctcaaa tcctgctggg
 541 acatggggtt cttggactgt tgaaggaacc taaccatctg gcatctccat ggcctctgca
 601 acaaaccccc cactttttttc tctccccccc cccccaggga gggcctgtgc tttgggcagc
 661 cacctgcccc tctcaggggt ttggagccag gcagggtcac agcagactgg aaacatcgga
 721 catgcatgtg caggctgggt gggagagacc gttctattcc tcagtgcaat tgtgttgctg
 781 aaagactacc tcgttcttgt ctttgtgtgt caccggggca actgtgtggg ggcggggatg
 841 ggggcagggt ggcagcacgc ccagtttggt atcaaaggtg ctacatctct gtgaaggggt
 901 ggggtgggaa ggaatttctg gtgctataga agctgagatg ctccctagac cagcaaatgt
 961 ttcttttgtt caaagtattt tttattcttt tttttttttt ttttaatgga tagggacttg
1021 tgtgaattgt tggggttttt tttttttttt tttttggttt tgtttttttt tgttttgttt
1081 tgtttttttt cctgaaggtg ctatttaaca agggagaaga gagtgcgggg acttcaccct
1141 gcccactctc tactctctct ccactcttct agttcctggg cctatctgat ctctctcttt
1201 cttctgaaac cctcccctct tgctgctgct ccctcccctc tgcctctctc ttggtctgtc
1261 ctgcatcagg gtttcagagc accactttcc aaagcacaaa acagttttta cccctgggct
1321 gggaggaaac aagagactct gtacctattt tgtatgtgta taataatttg agatgttttt
1381 aattattttg attgctggaa taaagcatgt ggaaatgacc caaaaaaaaa aaaaaaaaa
```

Supplementary Table 2. hsa-microRNA-29c-3p and mmu-microRNA-29c-3p have binding sites on collagen type I alpha 1
